# Microbial gradual shifts during the process of species replacement in Taihang Mountain

**DOI:** 10.3389/fmicb.2023.1158731

**Published:** 2023-04-05

**Authors:** Xiuping Liu, Wangming Zhou, Xinzhen Wang, Hongliang Wu, Wenxu Dong

**Affiliations:** ^1^Key Laboratory of Agricultural Water Resources, Hebei Key Laboratory of Soil Ecology, Center for Agricultural Resources Research, Institute of Genetics and Developmental Biology, Chinese Academy of Sciences, Shijiazhuang, China; ^2^School of Life Sciences, Anqing Normal University, Anqing, China

**Keywords:** species replacement, bacteria, fungi, microbial composition, indicator species, Taihang Mountain

## Abstract

**Introduction:**

Understanding microbial gradual shifts along species replacement can help elucidate the mechanisms driving secondary succession, and predict microbial responses to changing environments. However, how climate-induced species replacement alters microbial processes, and whether microbial shifts follow predictable assembly trajectories remain unclear.

**Methods:**

Using space-for-time substitution approach, we studied shifts in bacterial and fungal communities in the succession from *Leptodermis oblonga* to *Vitex negundo* var. *heterophylla* shrubland in Taihang Mountain.

**Results and Discussion:**

Species replacement, induced by climate related environmental change, significantly increased the above-ground biomass of shrublands, and TP and TK contents in topsoil. The succession from *L*. *oblonga* to *V*. *negundo* var. *heterophylla* communities resulted in the gradually replacement of cold-tolerant microbes with warm-affinity ones, and alterations of microbial communities involved in soil biogeochemical processes. Soil and plant variables, such as above-ground biomass, soil pH, total phosphorus, and total potassium, well explained the variations in microbial communities, indicating that the coordinated changes in plant communities and soil properties during secondary succession caused accompanied shifts in microbial diversity and composition.

## 1. Introduction

Soil microorganisms are an integral component of forest ecosystem, playing an essential role in regulating critical ecosystem processes such as primary production and nutrient cycling (de Gannes et al., 2016; Zhong et al., 2018). Changes in microbial communities occurring with secondary succession are closely related to shifts in ecological function (Heine et al., 2019; Zhong et al., 2019; Liu J. et al., 2020), and microbial diversity and composition are mainly mediated by plant traits and soil properties (Chai et al., 2019; Yan et al., 2020). Therefore, understanding shifts in microbial communities across successional stages could help illuminate the coupling relationships among plants, soil, and microbial community during the process of secondary succession (Zhong et al., 2018; Jiang et al., 2021).

Secondary succession, induced by replacement of dominant species, cause gradual shifts in plant community and soil properties, and thus resulting in microbial succession (Schmidt et al., 2014; Heine et al., 2019; Zhang et al., 2021). Previous studies have reported changes in and drivers of microbial diversity and composition along successional gradients in different ecosystems (Nacke et al., 2016; Szoboszlay et al., 2017; Ding et al., 2020; Wang G. et al., 2022). However, due to the inherent heterogeneity among different successional environments, bacteria and fungi may perform different or even opposite shift and reassemble patterns (Gao et al., 2015; Zhou et al., 2017; Li C. et al., 2018). Therefore, how species replacement alters microbial processes, and whether microbial shifts follow predictable assembly trajectories remain unclear (Schmidt et al., 2014; Chai et al., 2019). Furthermore, current studies mostly focused on microbial succession in different vegetation types (forest, grasslands, farmland, etc.) (Cui et al., 2019; Liu Y. et al., 2020), but neglected microbial gradual shifts during the process of species replacement. Still, it remains unclear whether microbial shift and reassemble smoothly during the process of vegetation succession (Liu J. et al., 2020).

Climate and environment changes are expected to affect plant communities and consequently microbial composition (Cregger et al., 2012; Mou et al., 2022). Shifts in plant communities, such as plant diversity, composition, and biomass, can alter litter inputs and root exudates, which in turn influence microbial diversity and its related microbial process (Bakker et al., 2014; Lange et al., 2015; Xu et al., 2020; Wang G. et al., 2022). Moreover, soil properties such as pH and nutrient concentrations can regulate microbial composition or metabolic diversity through their effects on enzyme kinetics and nutrient diffusion (Zhang et al., 2016; Han et al., 2021). In turn, the shifts in microbial composition can also influence plant diversity by regulating nutrient bioaccessibility and then altering plant dominance (Van Der Heijden et al., 2008; Philippot et al., 2013; Kyaschenko et al., 2017). Thus, vegetation succession is essentially the interaction among plant, soil, and microorganisms (He et al., 2008; Kielak et al., 2008), a better understanding shifts in microbial composition along species replacement could help delineate the mechanisms that drive secondary succession and predict how microbes will respond to changing environments (Fierer et al., 2010; Jiang et al., 2021).

**Figure 1:**
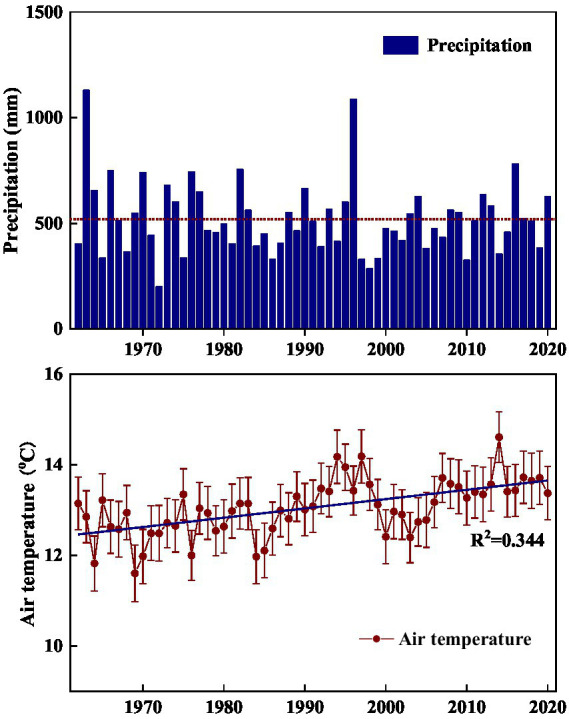
Annual variability of precipitation **(A)** and air temperature **(B)** in the hilly area of Taihang Mountain from 1962 to 2020. Precipitation is annual total and air temperature is annual average.

The current study was conducted in the hilly area of Taihang Mountain. In this region, before 1970s, large-scale human-induced deforestation such as clear-cutting, tilling, logging, and grazing has severely destroyed the original forest vegetation, and converted it into degraded shrub-herb communities (Calvo et al., 2002; Liu et al., 2012; Baudena et al., 2020). Starting from 1980s, a series of vegetation conservation projects such as banning grazing and returning marginal cropland to forest or grassland, were implemented to limit human disturbance, avoid soil degradation, and promote vegetation recovery (Liu et al., 2012; Guo et al., 2021). In addition, according to meteorological data of the study site, air temperatures exhibited a gradually increasing trend in the past 50 years (Figure 1). As affected by climate related environmental change, the vegetation in the area has experienced transition from perennial herbs to shrub-herb and then shrub communities, and shrublands have undergone gradual succession from *Vitex negundo* var. *heterophylla* and *Leptodermis oblonga* co-dominate to *V*. *negundo* var. *heterophylla* dominant communities (data not published). As succession progresses, community height, cover, and aboveground biomass increased gradually, while species diversity decreased generally (data not published). Thus, it offers an ideal landscape to investigate how the environmentally induced species replacement alters microbial processes. Here, we selected three successional stages (*V*. *negundo* var. *heterophylla* shrubland Orthodonic, *V*. *negundo* var. *heterophylla* and *L*. *oblonga* co-dominate shrubland (VLS), and *L*. *oblonga* shrubland (LS)) to (1) elucidate how microbial diversity and composition shifts along species replacement; (2) identify the indicator species of shrubland during the process of secondary succession; and (3) determine which factors are closely related to shifts in microbial community.

## 2. Materials and methods

### 2.1. Site description

This study was conducted in the Niujiazhuang Catchment (area: 9.3 km^2^) of middle Taihang Mountain, China (114°15′50″ E, 37°52′44″ N). Elevation across the study area ranges from 247 to 1,040 m a.s.l. and slope varies from 20 to 45°. This region has a typical temperate continental monsoon climate with wet warm summers and cool dry winters. Annual average precipitation from 1962 to 2020 is about 519 mm, 74% of which falling between June and September, and monthly mean air temperature ranges from −3.07°C in January to 26.8°C in July (Figure 1). The soil type is mainly highly-weathered mountainous cinnamon, classified as Ustalf (Liu et al., 2014). These soils are fertile and well-structured on shady slopes and skeletal and rocky on dry sunny slopes.

The original forests in the area have been severely destroyed and replaced by degraded shrub-herb communities. To better understand the succession processes of shrub-herb communities, we established 144 2 m × 2 m permanent plots in the Niujiazhuang Catchment, and conducted vegetation and soil census in 1986 and 2008, and in 2020, permanent plots were grouped by vegetation type and geographic location, and 34 plots with expanded area of 16 m^2^ were selected for re-census. In 1986, most plots were dominated by perennial herbs, with a high number of species composition and abundance, and low number of aboveground biomasses (Liu et al., 2011). From 1986 to 2008, along the decrease of herbaceous species, abundance, and biomass, the proportion of perennial herbs declined in favor of shrubs dominated by *V*. *negundo* var. *heterophylla* and *Ziziphus jujuba* var. *spinosa* on south-facing slope, and *L*. *oblonga* on north-facing slope (Liu et al., 2012). After a few years, in 2020, *V*. *negundo* var. *heterophylla* has become the most dominant species in the hilly area of Taihang Mountain, as it had highest importance value, height, cover, and biomass in all layers (data not published).

### 2.2. Vegetation investigation and soil sampling

Due to lack long-term monitoring of microbial dynamics, we used space-for-time substitution to analyze how microbial shifts as plants transitioned from LS to VLS and then VS. Along with vegetation census in September 2020, according to dominant species, soil properties, and spatial distribution, a total of 20 plots (8 VS, 6 VLS, and 6 LS) were selected to represent the typical successional stages during the process of species replacement. Except for slope aspect (VS often grows on sunny slope, while VLS and LS concentrate on shady slope), all plots have similar geographical distribution, including elevations, slope gradient, slope position, and soil type. In each plot, the species, number, height, cover, and aboveground biomass of shrubs and herbs were recorded, and topographic factors such as elevation, slope gradient, slope aspect, and slope position were also recorded. The aboveground biomass of shrubs and herbs were measured by expanding the sampling domain to neighboring areas of the plot. The plant samples were oven-dried at 80°C to a constant weight and weighed for dry matter.

In each plot, soil samples were collected from the top 20 cm using a 5 cm diameter soil auger. After removing visible plant roots, stones, litter, and debris, nine soil cores were collected along an S-shaped transect and then mixed together to form three composite sample. Thus, a total of 60 soil samples were collected (20 plots × 3 replicates). Each soil sample was divided into two subsamples after removing visible plant roots, stones, litter, and debris. One subsample was immediately stored at −80°C for DNA analysis, and the other was air-dried for physicochemical analysis.

### 2.3. Soil physicochemical properties

Soil density (SD) at 0–20 cm depth was estimated in undisturbed samples, collected with cylindrical stainless steel rings (100 cm^3^), from three samples per plot. Soil pH was determined in 1:2.5 soil/water suspension using a pH meter (PHS-3C, Shanghai, China) (Bao, 2000). Soil organic matter (SOM) was determined by the Walkley-Black potassium dichromate oxidation method after H_2_SO_4_-HCLO_4_ digestion (Nelson and Sommers, 1982). Total nitrogen (TN) was determined by the Kjeldahl method with an automated Kjeldahl apparatus (Kjeltec 8,400, Foss, Sweden) (Bremner, 1996). Total phosphorus (TP) was digested by perchloric acid and determined by the molybdate colorimetric method with a UV spectrophotometer (UV-2450, Shimadzu, Japan) (O’Halloran and Cade-Menun, 2006). Total potassium (TK) was determined by flame atomic absorption spectrophotometer (Analytikjena, Germany).

### 2.4. Soil DNA extraction, polymerase chain reaction amplification, and illumina sequencing

Soil DNA was extracted for three sub-samples from 0.5 g of soil using FastDNA Spin Kit (MP Biomedicals, Cleveland, United States) following the manufacturer’s instructions. DNA concentration was determined with a NanoDrop ND-2000 spectrophotometer (NanoDrop Thermo Scientific, Wilmington, DE, United States), and DNA quality was evaluated by 1% agarose gel electrophoresis. Polymerase chain reaction (PCR) amplification of bacterial 16S rRNA V4-V5 region and fungal ITS1 region were conducted using the 515F/907R and ITS1F/ITS2R primer sets, respectively (Peng et al., 2019; Zhong et al., 2019). Detailed protocols for PCR amplification have been described in previous reports (Song et al., 2018; Zhao et al., 2019; Zhong et al., 2019). Each DNA extract was amplified in three replicates and then were mixed into one PCR product. After amplification, each mixed gene was detected by 2% agarose gel electrophoresis, purified with AxyPrepDNA purification kit (Axygen, United States), and quantified by Quantus™ Fluorometer (Promega, Madison, WI, United States) (Zhang et al., 2016; Liu et al., 2018). Subsequently, all amplicons were sequenced on the Illumina Miseq platform (Personal Biotechnology Co., Ltd., Shanghai, China). Approximately 50,884 and 14,868 high quality sequences per sample with an average length of 363 and 447 bp were obtained for bacteria and fungi, respectively.

Raw sequencing data were demultiplexed, quality-filtered, and analyzed using QIIME (Caporaso et al., 2010). After reads<50 bp and any unresolved nucleotides were discarded, noise filtering and chimera removal were performed using USEARCH (Edgar, 2010; Edgar et al., 2011), and high-quality sequences were assigned to operational taxonomic units (OTUs) using Silva and Unite databases with a similarity threshold of 97% (Caporaso et al., 2010). Finally, a total of 9,025 bacterial and 6,344 fungal OTUs were detected after trimming, assembly, and quality filtering.

### 2.5. Statistical analyses

Microbial community diversity (shannon), richness (Chao1), and rarefaction curve were performed using Mothur version 1.30.2. The Kolmogorov–Smirnov test was used to check the data distribution, and all data sets met the normality assumption for one-way analysis of variance (ANOVA). ANOVA followed by least significant difference (LSD) multiple comparison (*p* < 0.05) was used to assess the differences in plant traits (cover, height, and aboveground biomass), soil properties (pH, SOM, TN, TP, TK, and SD), and microbial alpha diversity (Shannon and Chao1 indices) and abundances among successional stages. Venn diagrams of shared and unique OTUs among different successional stages were performed using R-package VennDiagram. Indicator species analysis in the R indicspecies package was used to identify OTUs that were significantly associated with three successional stages, and discuss their potential as indicator species. Principal component analysis (PCA) was performed to explain the relationship between microbial genes (OTU) and environment variables (pH, SOM, TN, TP, TK, SD, cover, height, and aboveground biomass) during succession. ANOVA analysis was performed using SPSS 19 for Windows (SPSS Inc., Chicago, United States), diversity index and rarefaction curve were calculated using Mothur software,^1^ PCA and indicator species analysis were conducted in R software package v4.2.1.^2^

## 3. Results

### 3.1. Plant and soil properties

**Figure 2:**
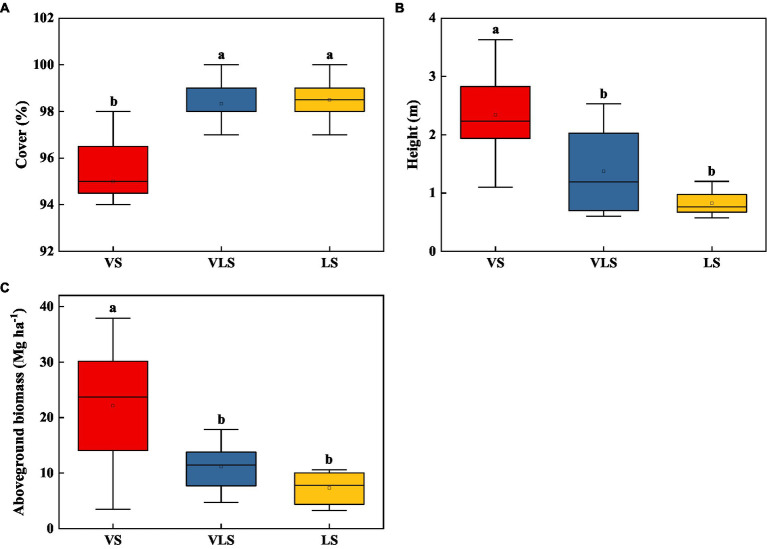
Changes in height **(A)**, cover **(B)**, and aboveground biomass **(C)** of shrub-herb communities across successional stages. Different letters indicate significant differences at *p* < 0.05 according to ANOVA. VS, *Vitex negundo* var. *heterophylla* shrubland; VLS, *Vitex negundo* var. *heterophylla* and *Leptodermis oblonga* shrubland; LS, *Leptodermis oblonga* shrubland.

In 2020, the mean height, cover, and aboveground biomass of VS, VLS, and LS were 2.19 m, 97.2%, and 15.7 Mg ha^−1^ (Figure 2). VS exhibited significantly higher community height and aboveground biomass compared with VLS and LS, who showed higher community cover (*p* < 0.05). However, no significant differences in plant traits were detected between VLS and LS (*p* > 0.05) (Figure 2).

**Figure 3:**
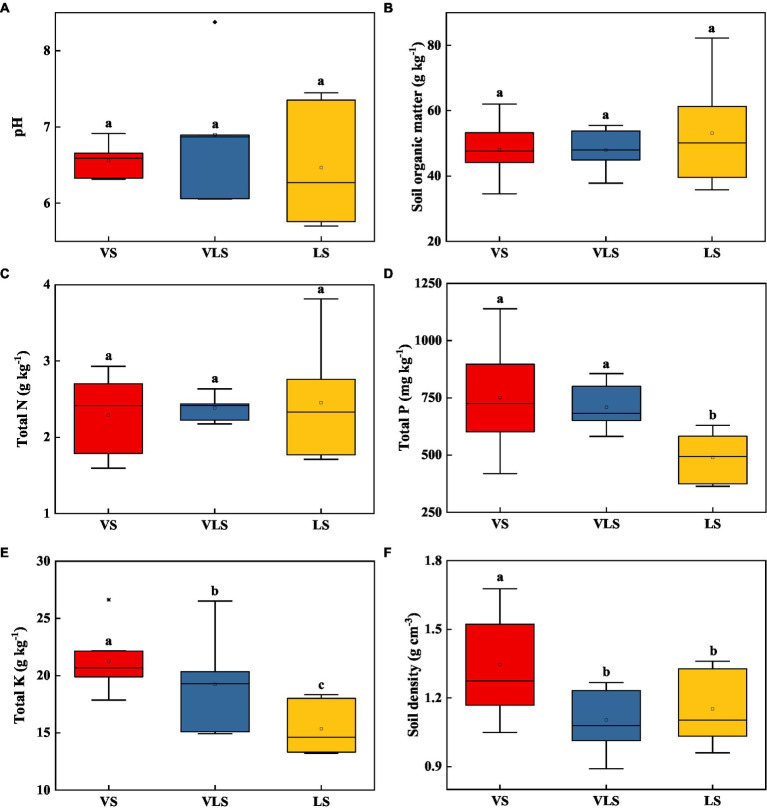
Changes in soil pH **(A)**, soil organic matter **(B)**, total N **(C)**, total P **(D)**, total K **(E)**, and soil density **(F)** in topsoil (0–20 cm) across successional stages. Different letters indicate significant differences at *p* < 0.05 according to ANOVA. VS, *Vitex negundo* var. *heterophylla* shrubland; VLS, *Vitex negundo* var. *heterophylla* and *Leptodermis oblonga* shrubland; LS, *Leptodermis oblonga* shrubland.

Among three successional stages, there were no significant differences regarding soil pH, SOM, and TN (*p* > 0.05) (Figure 3). VS and VLS showed significantly higher TP than LS, and VS exhibited higher SD relative to VLS and LS (*p* < 0.05) (Figure 3). Compared to VS, VLS showed a lower TK, while compared to LS they displayed higher TK (*p* < 0.05) (Figure 3).

### 3.2. Diversity and composition of microbial communities

**Figure 4:**
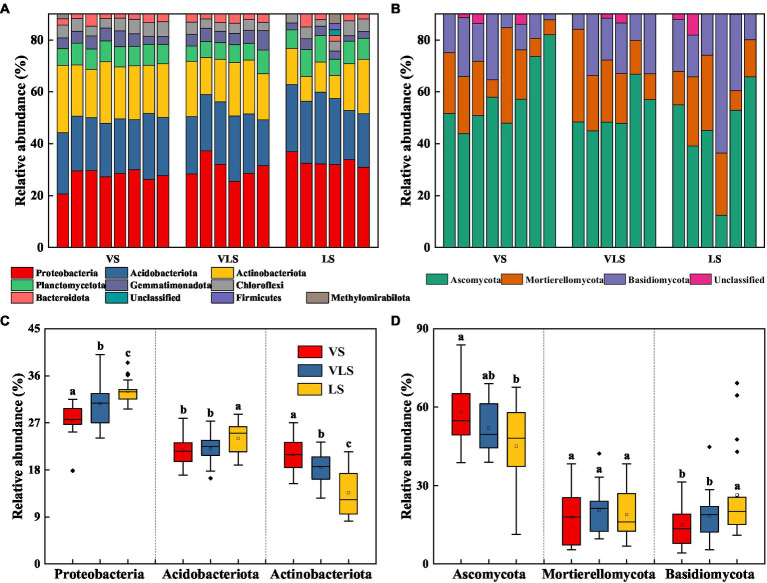
Relative abundance of bacteria **(A)** and fungi **(B)** at the phylum level, and relative abundance of dominant bacterial **(C)** and fungal **(D)** phyla. Phyla with low abundances (<2%) were not shown. Different letters indicate significant differences at *p* < 0.05 according to ANOVA. VS, *Vitex negundo* var. *heterophylla* shrubland; VLS, *Vitex negundo* var. *heterophylla* and *Leptodermis* *oblonga* shrubland; LS, *Leptodermis oblonga* shrubland.

Proteobacteria (30.1%) was the most dominant bacterial phylum in all samples, followed by Acidobacteriota (22.5%) and Actinobacteriota (18.1%) (Figure 4). The relative abundance of Proteobacteria and Acidobacteriota decreased, while that of Actinobacteriota increased with succession (*p* < 0.05) (Figure 4). The fungal communities were dominated by Ascomycota (52.4%), Mortierellomycota (19.0%), and Basidiomycota (19.5%) across three successional stages (Figure 4). There were no significant differences in relative abundance of Mortierellomycota (*p* > 0.05), but significant increases in Ascomycota abundance and decreases in Basidiomycota abundance as succession progresses (*p* < 0.05) (Figure 4).

**Figure 5:**
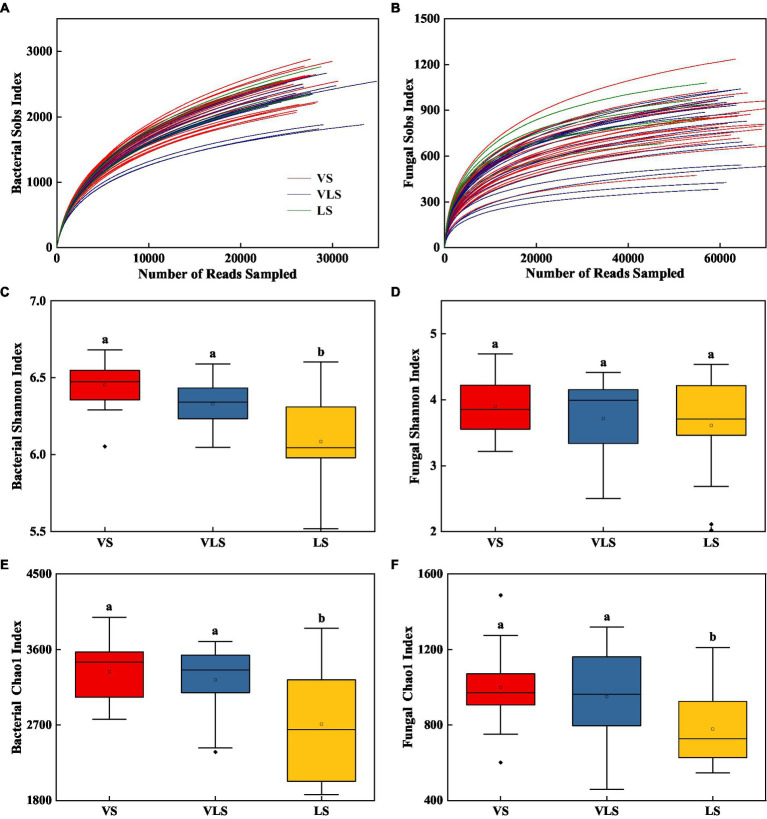
Changes in microbial diversity of bacteria and fungi across successional stages. **(A)** bacterial rarefaction curves, **(B)** fungal rarefaction curves, **(C)** Shannon index of bacteria, **(D)** Shannon index of fungi, **(E)** Chao 1 index of bacteria, **(F)** Chao 1 index of fungi. Different letters indicate significant differences at *p* < 0.05 according to ANOVA. VS, *Vitex negundo* var. *heterophylla* shrubland; VLS, *Vitex negundo* var. *heterophylla* and *Leptodermis oblonga* shrubland; LS, *Leptodermis oblonga* shrubland.

Rarefaction curves suggested that the overall bacterial and fungal OTUs were well captured at the current sequence depth (Figure 5). VS and VLS showed higher bacterial diversity than LS (*p* < 0.05), but there were no significant differences in fungal diversity among different successional stages (*p* > 0.05) (Figure 5). Soils in VS and VLS exhibited higher bacterial and fungal richness, compared to the soils in LS (*p* < 0.05) (Figure 5).

**Figure 6:**
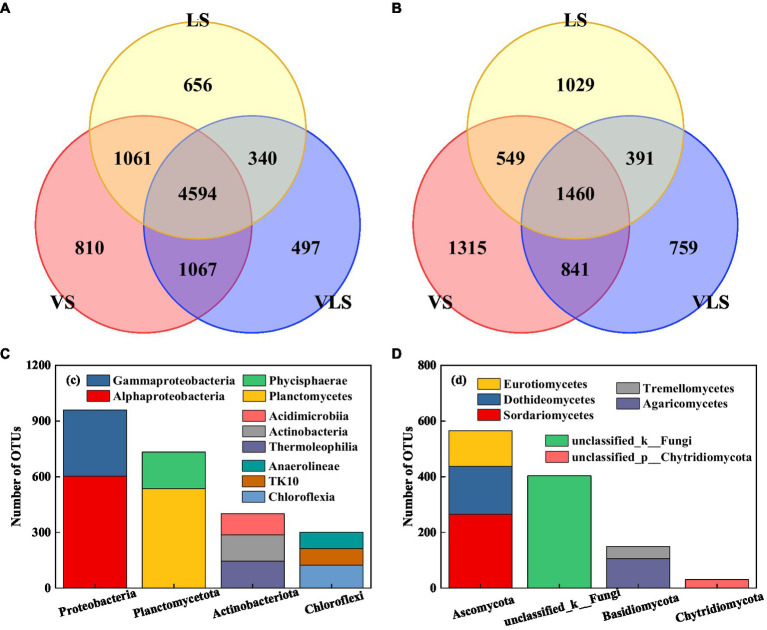
Venn diagrams and core OTUs of bacteria and fungi. **(A)** venn diagram of bacteria, **(B)** venn diagram of fungi, **(C)** core OTUs of bacteria, and **(D)** core OTUs of fungi. VS, Vitex negundo var. heterophylla shrubland; VLS, Vitex negundo var. heterophylla and *Leptodermis* oblonga shrubland; LS, *Leptodermis* oblonga shrubland. VS, *Vitex negundo* var. *heterophylla* shrubland; VLS, *Vitex negundo* var. *heterophylla* and *Leptodermis oblonga* shrubland; LS, *Leptodermis oblonga* shrubland.

Venn diagrams indicated that the three successional stages shared 50.9% bacterial and 23.0% fungal OTUs (Figure 6). The core bacterial microbiome was dominated by Proteobacteria and Planctomycetota, among which Alphaproteobacteria (34.4%), Planctomycetes (30.6%), and Gammaproteobacteria (20.3%) were identified as the most predominant classes (Figure 6). Most of the core fungal microbiome belonged to Ascomycota, and 75.8% of these taxa belonged to Sordariomycetes, Dothideomycetes, and Eurotiomycetes (Figure 6).

### 3.3. Indicator species of successional stages

**Figure 7:**
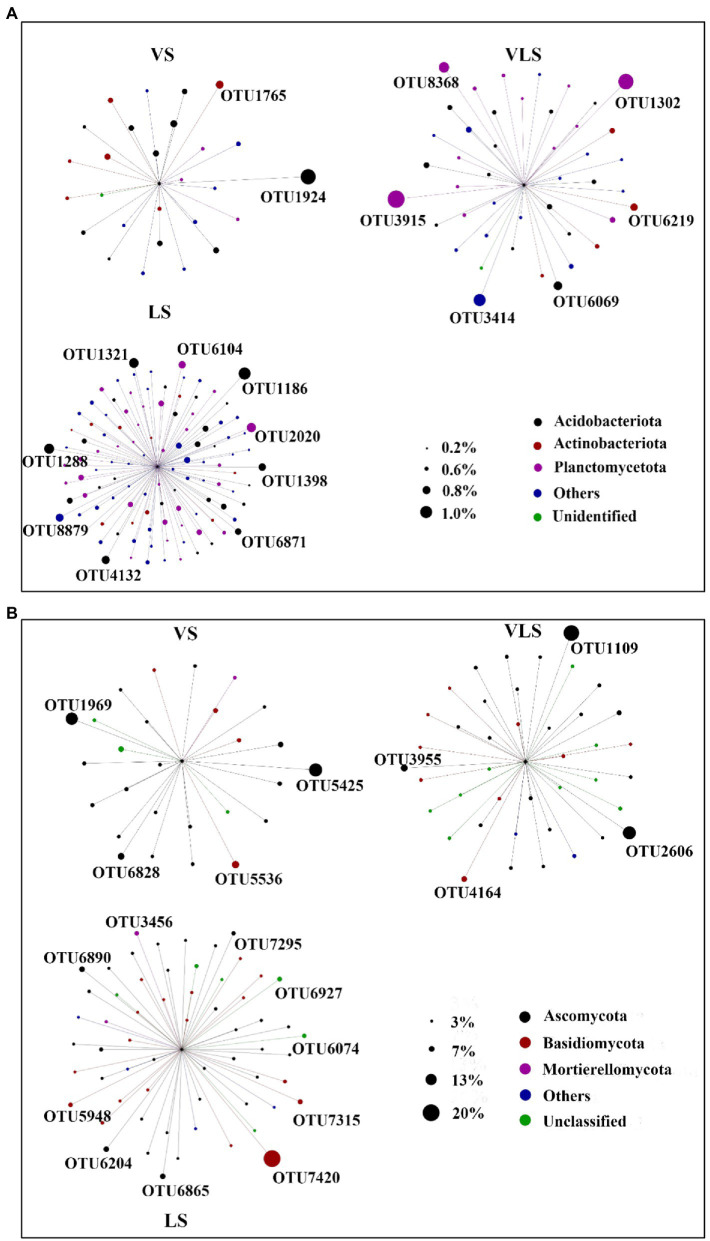
Indicator species by treatment regime. Circles represent OTUs, and the size of each circle represents its relative abundance. OTUs with low abundances (bacteria<0.1%, and fungi<0.3%) were not shown. VS, *Vitex negundo* var. *heterophylla* shrubland; VLS, *Vitex negundo* var. *heterophylla* and *Leptodermis oblonga* shrubland; LS, *Leptodermis oblonga* shrubland.

Indicator species of bacterial and fungal community varied among three successional stages (Figure 7; Supplementary Table S1). For bacteria, the most abundant OTU in VS was assigned to Vicinamibacteraceae, a typically aerobic heterotroph that can survive in arid environments (Huber and Overmann, 2018; Wang K. et al., 2022). Whereas, the most abundant OTU in LS belonged to Acidobacteriales, an order comprising aerobic or facultatively anaerobic, and mostly acidophilic and mesophilic bacterium (Kuramae and de Assis Costa, 2019). In addition, OTU3915, together with OTU1302 and OTU3414, were the indicator species in VLS, and performed major roles in soil nitrogen and phosphorus cycles (Supplementary Table S1). Correspondingly, for fungi, the most abundant OTU in VS belonged to *Pleiochaeta*, a genus with pathogenic members that could cause leaf spots on legumes (Marin-Felix et al., 2017). However, one of the most abundant OTUs in VLS was classified as a member of the phylum Ascomycota with a relative abundance of 11.0%. Furthermore, the most abundant OTU in LS was OTU7420, and belonged to Basidiomycota, the major degraders of different components in wood (Taylor et al., 2015).

### 3.4. Shifts in microbial community

**Figure 8:**
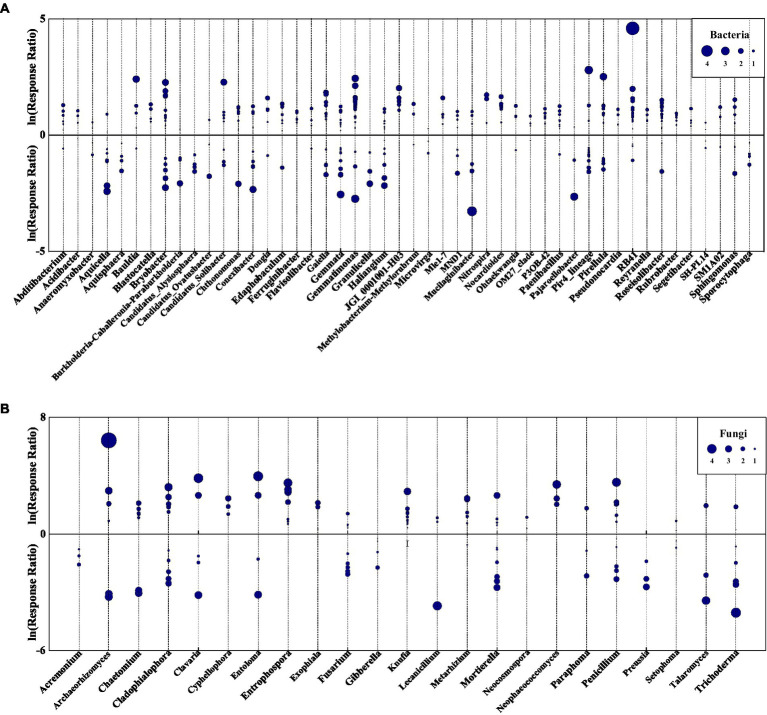
Log response ratio of bacterial **(A)** and fungal **(B)** genera in *VS* and VLS plots relative to LS plots. Circles represent OTUs with *p* < 0.05, and the size of each circle represents its relative abundance. VS, *Vitex negundo* var. *heterophylla* shrubland; VLS, *Vitex negundo* var. *heterophylla* and *Leptodermis oblonga* shrubland; LS, *Leptodermis oblonga* shrubland.

Eurythermal bacterial OTUs growing across a large temperature gradient increased in abundance due to species replacement, however, some stenothermal species with narrow growth temperature ranges declined with succession (Figure 8; Supplementary Figures S1, S2; Supplementary Tables S2, S3). Among indicator OTUs showing increased relative abundance following species replacement were acidophilic bacteria, such as *Acidibacter* and *Catenulispora*, pathogenic taxa, such as *Enterobacter* and *Legionella*, heat-tolerant taxa, such as *Legionella* and *Rhodovastum*, and some metabolism bacteria (Supplementary Table S2). The bacterial groups that declined with succession were mainly those associated with nitrogen cycling and biodegradation of organic pollutants, as well as a few psychrotolerant, acidophilic, and pathogenic bacteria (Supplementary Table S3).

Fungal indicator OTUs were more likely to decline with succession than bacterial ones (Figure 8; Supplementary Figures S3, S4; Supplementary Tables S4, S5). Ascomycota was the most abundant fungi phyla in all samples, and a majority of indicator OTUs in Ascomycota declined due to species replacement, while a minority expanded with succession (Supplementary Figures S3, S4; Supplementary Tables S4, S5). Among indicator OTUs showing declined relative abundance were endophytic taxa, such as *Apiospora* and *Aureobasidium*, saprobic taxa, such as *Chaetosphaeria* and *Endophragmiella*, and some pathogenic fungi (Supplementary Figure S3; Supplementary Table S4). The fungal groups that increased with succession were mainly rock-inhabiting, nematode-trapping, endophytic, and pathogenic species (Supplementary Figure S4; Supplementary Table S5).

### 3.5. Key factors driving the shifts in microbial community

**Figure 9:**
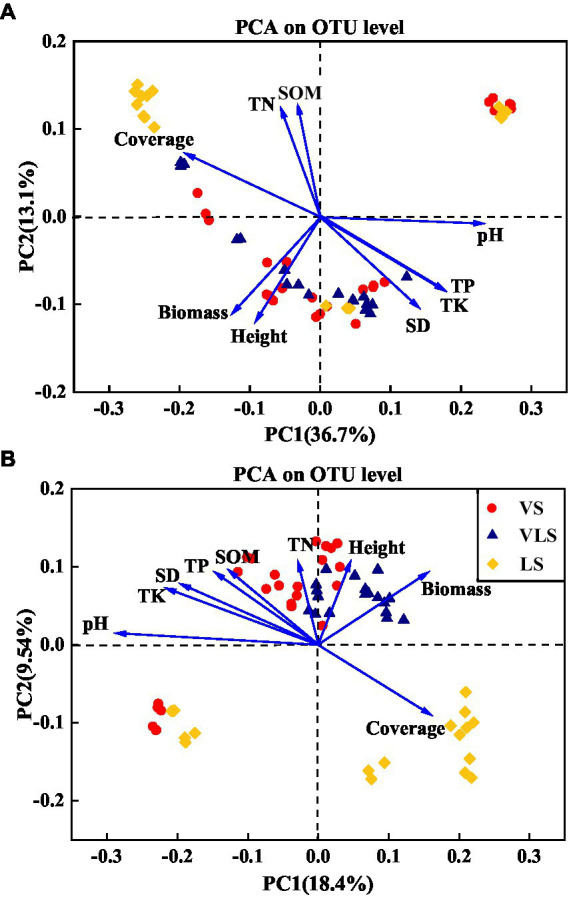
Principal component analysis (PCA) of the relationship between the bacterial **(A)**, fungal **(B)** community composition and environment variables. SOM: soil organic matter; TN: total N; TP: total P; TK: total K; SD: soil density; Biomass: community aboveground biomass; Height: community height; Cover: community cover. VS, *Vitex negundo* var. *heterophylla* shrubland; VLS, *Vitex negundo* var. *heterophylla* and *Leptodermis oblonga* shrubland; LS, *Leptodermis oblonga* shrubland.

According to the PCA results, the shifts in microbial community composition across successional stages were strongly associated with soil properties and plant traits (Figure 9). Soil pH, TK, and aboveground biomass significantly affected bacterial community composition, it could explain 49.8% of bacterial community variation (Figure 9). For fungal communities, soil pH, TK, aboveground biomass, and TP were best suited to explain the shifts in microbial composition, and explained 27.9% of the total variability of fungal community dynamics (Figure 9).

## 4. Discussion

Secondary succession in shrub-herb communities following disturbance has commonly been shown to directly alter species composition and ecosystem functions (Heine et al., 2019; Yan et al., 2020), and such changes in turn affect soil biochemical processes in degraded environments (Zhao et al., 2019; Jiang et al., 2021). In our results, during the process of species replacement, the aboveground biomass of shrubland increased over 3 times, from 7.3 Mg ha^−1^ in LS to 22.2 Mg ha^−1^ in VS (Figure 2), similar to the results of Li Q. et al. (2018) and Ma and Wang (2020). Aboveground biomass increased significantly in the presence of *V*. *negundo* var. *heterophylla*, indicating that these shrublands in the hilly area of Taihang Mountain are important carbon pools, and if all the established shrublands in this area are eventual replaced by *V*. *negundo* var. *heterophylla*, it will have great potential for carbon sequestration. These changes in plant community due to species replacement altered the distribution of soil nutrients, especially TP and TK (Figure 3; Feng et al., 2007; Sullivan et al., 2019; Segura et al., 2020). Possible explanations for the increased TP and TK with succession can be ascribed to plant species and local topographic features (Zhu et al., 2016; Jucker et al., 2018). The high morphological plasticity of *V*. *negundo* var. *heterophylla* (Moreira et al., 2003; Wang et al., 2017), allows it to use nutrients of fractured rocks on barren land, and facilitates P and K translocation from deeper soil layers to topsoil (Sullivan et al., 2019; Segura et al., 2020). Altogether, the coordinated changes in vegetation and soil highlight the importance of above- and belowground linkages as succession progresses (Roy-Bolduc et al., 2016).

The coordinated changes in plant and soil during secondary succession also caused accompanied shifts in microbial diversity and composition (Zhong et al., 2018; Chai et al., 2019; Zhao et al., 2019). As expected, our results showed that aboveground biomass significantly influenced microbial community composition across successional stages (Figure 9), consistent with previous findings (Chen et al., 2016; Zhang et al., 2016; Kyaschenko et al., 2017). This result may be ascribed to the fact that increased aboveground biomass with succession could accelerate accumulation of plant-derived resources and nutrients for microbial growth (Kardol et al., 2006; Lange et al., 2015). In addition, belowground soil properties, such as soil pH and nutrient concentrations, have also been identified as potential ecological drivers for shaping soil microbial processes (Figure 9; Kaiser et al., 2010; Hu et al., 2014). In this regard, large number of studies have confirmed the effects of soil pH on microbial community structure and function (Siciliano et al., 2014; Tripathi et al., 2015; Lu et al., 2022), and demonstrated that the differences in microbial community could be explained primarily by the variation in soil pH (Zhang et al., 2018; Chai et al., 2019). Moreover, we also found that TP and TK were significantly correlated with microbial community structure (Figure 9). Soil phosphorus and potassium were mainly derived from parent material, along the depleted and fixed in plants and animal tissues, their concentrations may restrict microbial growth and metabolisms (Zhao et al., 2013; Hou et al., 2017; Song et al., 2018). Therefore, both plant and soil properties shift driven by vegetation succession could structure soil microbial communities (Koyama et al., 2018).

Species replacement, induced by climate related environmental change, resulted in a gradually replacement of cold-tolerant microbes with warm-affinity ones (Figure 8; Supplementary Figures S1, S2). The relative abundance of bacteria growing optimally above 30°C increased (Figure 8; Pagnier et al., 2010; Falagán and Johnson, 2014; Percival and Williams, 2014), and the indicator species in VS can survive in environments where water is extremely scarce (Huber and Overmann, 2018; Wang K. et al., 2022). The consistent increases in warm-affinity taxa along species replacement reflected a high tolerance for drought and heat stress of *V*. *negundo* var. *heterophylla* for survival in harsh environment, so it has potential to expand its range under climate warming (Du et al., 2014; Li et al., 2017; Wang et al., 2017). In addition, species replacement altered the relative abundance of several bacterial groups involved in soil biogeochemical processes (Supplementary Figures S1, S2). These included declining populations of *Asticcacaulis* (Poindexter, 2015) and *Dongia* (Liu et al., 2010), and increasing abundances of *Novosphingobium* (Kumar et al., 2022) and *Rhizobacter* (Goto, 2015), previously identified as major degraders of organic compounds. Moreover, species replacement also produced significant expansion in *Microlunatus* (Hanada and Nakamura, 2015) and *Rhodovastum* (Okamura et al., 2009), and decline in *Dyella* (Xie and Yokota, 2005) and *Rivibacter* (Stackebrandt et al., 2009), which was previously linked to soil nutrient cycles. However, populations of *Enterobacter* (Iversen, 2014) and *Rhizorhapis* (Francis et al., 2014) also increased following species replacement, a group of animal or plant pathogens. Therefore, secondary succession, induced by the replacement of dominant species, resulted in significant changes in soil environment and bacterial microbial community.

Ascomycota was the most abundant phylum of fungi, the decline of their dominant taxa was consistent with the finding of Zhang et al. (2018) and Chai et al. (2019). Generally, members of Ascomycota are dominant in stressful environments (Tripathi et al., 2016), the decline of dominant taxa indicated that soil ecological environments were improved through vegetation succession (Dong et al., 2016; Chai et al., 2019). In contrast, the overall relative abundance of Ascomycota increased with succession (Figure 4), similar to the observations in fire-affected and exposed soil environments (Taş et al., 2014; Wilhelm et al., 2017), this pattern likely reflected the increase of rock-inhabiting and some other extremotolerant fungi (thermophilic, desiccation-tolerant, etc.) during gradual expansion of *V*. *negundo* var. *heterophylla* toward harsh environment (Morgenstern et al., 2012; Egidi et al., 2014; Hubka et al., 2014). Notably, the nematophagous subset of Ascomycota, including *Dactylellina*, *Purpureocillium*, and *Pochonia*, that expanded in most plots following species replacement have all been reported in mountain environments and forest soils (Li et al., 2006; Deng et al., 2020; Gouveia et al., 2022). Numerous nematophagous fungi can immobilize and digest nematodes, and are thought to be important in regulating entomopathogenic nematode populations in the field (Wang et al., 2006; Willett et al., 2017). All these results indicated that various fungal microbial communities exhibited different adaptions to shifts in environmental conditions during secondary succession (Schmidt et al., 2014; Alfaro et al., 2017).

## 5. Conclusion

Secondary succession from *L*. *oblonga* to *V*. *negundo* var. *heterophylla* shrubland in Taihang Mountain significantly increased the above-ground biomass of shrublands, and TP and TK contents in topsoil. Species replacement, induced by climate related environmental change, resulted in the gradually replacement of cold-tolerant microbes with warm-affinity ones, and alterations of microbial communities involved in soil biogeochemical processes. Soil and plant variables, such as above-ground biomass, soil pH, TP, and TK, well explained the variations in microbial communities. Altogether, the coordinated changes in plant communities and soil properties during secondary succession caused accompanied shifts in microbial diversity and composition.

## Data availability statement

The original contributions presented in the study are included in the article/Supplementary materials, further inquiries can be directed to the corresponding author.

## Author contributions

XL and WZ: investigation, formal analysis, and writing–original draft. XW and HW: methodology, data curation, and writing–review and editing. WD: resources, supervision, and project administration. All authors contributed to the article and approved the submitted version.

## Funding

This research was supported by the Key Research and Development Program of Hebei Province (22326412D), the “Strategic Priority Research Program” of the Chinese Academy of Sciences (XDA26040103, XDA28020303), the National Key Research and Development Program of China (2021YFD1901104), and the Natural Science Foundation of Hebei Province (D2021503009).

## Conflict of interest

The authors declare that the research was conducted in the absence of any commercial or financial relationships that could be construed as a potential conflict of interest.

## Publisher’s note

All claims expressed in this article are solely those of the authors and do not necessarily represent those of their affiliated organizations, or those of the publisher, the editors and the reviewers. Any product that may be evaluated in this article, or claim that may be made by its manufacturer, is not guaranteed or endorsed by the publisher.

## Footnotes

^1^ https://mothur.org/wiki/calculators/

^2^ http://www.r-project.org/
