# Supplementary material for: Microbial gradual shifts during the process of species replacement in Taihang Mountain
Source: Front Microbiol. 2023 Apr 5;14:1158731. doi: 10.3389/fmicb.2023.1158731 (PMC10113637; doi:10.3389/fmicb.2023.1158731)
Supplement: Supplementary file 1 [file Data_Sheet_1.docx]

Fig S1 Abundance profiles of bacterial genera in Proteobacteria (a) and Actinobacteriota (b) with increased abundance along species replacement. VS: *Vitex* *negundo* var. *heterophylla* shrubland; VLS: *Vitex* *negundo* var. *heterophylla* and *Leptodermis* *oblonga* shrubland; LS: *Leptodermis* *oblonga* shrubland.

Fig S2 Abundance profiles of bacterial genera in Proteobacteria with decreased abundance along species replacement. VS: *Vitex* *negundo* var. *heterophylla* shrubland; VLS: *Vitex* *negundo* var. *heterophylla* and *Leptodermis* *oblonga* shrubland; LS: *Leptodermis* *oblonga* shrubland; ANP: Allorhizobium-Neorhizobium-Pararhizobium-Rhizobium; BCP: Burkholderia-Caballeronia-Paraburkholderia; CA: Candidatus_Alysiosphaera; CB: Candidatus_Berkiella; CO: Candidatus_Ovatusbacter.

Fig S3 Abundance profiles of fungal genera in Ascomycota with decreased abundance along species replacement. VS: *Vitex* *negundo* var. *heterophylla* shrubland; VLS: *Vitex* *negundo* var. *heterophylla* and *Leptodermis* *oblonga* shrubland; LS: *Leptodermis* *oblonga* shrubland.

Fig S4 Abundance profiles of fungal genera in Ascomycota with increased abundance

along species replacement. VS: *Vitex* *negundo* var. *heterophylla* shrubland; VLS: *Vitex* *negundo* var. *heterophylla* and *Leptodermis* *oblonga* shrubland; LS: *Leptodermis* *oblonga* shrubland.
