# Supplementary material for: Microbial gradual shifts during the process of species replacement in Taihang Mountain
Source: Front Microbiol. 2023 Apr 5;14:1158731. doi: 10.3389/fmicb.2023.1158731 (PMC10113637; doi:10.3389/fmicb.2023.1158731)
Supplement: Supplementary file 2 [file Table_1.docx]

**Table S1** Indicator species of bacteria and fungi across successional stages. VS: *Vitex* *negundo* var. *heterophylla* shrubland; VLS: *Vitex* *negundo* var. *heterophylla* and *Leptodermis* *oblonga* shrubland; LS: *Leptodermis* *oblonga* shrubland.

| **Bacteria** | **OTU** | **Indicator**  **value** | **P** | **Relative abundance (%)** | **Taxonomy** | **Description** |
| --- | --- | --- | --- | --- | --- | --- |
| VS | OTU1924 | 0.373 | 0.012 | 1.262 | f__Vicinamibacteraceae | Members of the Vicinamibacteraceae are aerobic, neutrophilic, psychrotolerant to mesophilic bacteriums (Huber and Overmann, 2018) |
|  | OTU1765 | 0.493 | 0.001 | 0.555 | g__Streptomyces | Performing major roles in soil carbon cycles (Stone et al., 2021) |
|  | OTU6013 | 0.405 | 0.007 | 0.470 | g__RB41 |  |
| VLS | OTU3915 | 0.320 | 0.037 | 1.681 | g__Bradyrhizobium | Gram-negative nitrogen-fixing bacteria(Beeckmans and Xie, 2015) |
|  | OTU1302 | 0.539 | 0.001 | 1.489 | g__Ellin6067 | ammonia-oxidizing bacteria (Xia et al., 2005) |
|  | OTU3414 | 0.610 | 0.001 | 1.119 | f__Gemmatimonadaceae | Gram-negative, aerobic, polyphosphate-accumulating micro-organism (Zhang et al., 2003) |
|  | OTU8368 | 0.329 | 0.025 | 0.924 | g__Ramlibacter | Gram-negative, strictly aerobic, bacterium (Lee et al., 2014) |
|  | OTU6069 | 0.322 | 0.039 | 0.739 | g__RB41 |  |
|  | OTU6219 | 0.409 | 0.003 | 0.603 | g__Arthrobacter | Arthrobacter is a genus of obligate aerobes bacteria, they can degrade unusual and polymeric compounds and play an important role in biodegrading agrochemicals and pollutants (Gobbetti and Rizzello, 2014) |
| LS | OTU1186 | 0.361 | 0.001 | 1.036 | o__Acidobacteriales | Gram-negative, aerobic or facultatively anaerobic and mostly mesophiles bacterium (Kuramae and de Assis Costa, 2019) |
|  | OTU1288 | 0.534 | 0.001 | 0.848 | g__Terracidiphilus | Performing major roles in soil carbon cycles (García-Fraile et al., 2016) |
|  | OTU1321 | 0.336 | 0.027 | 0.812 | o__Acidobacteriales |  |
|  | OTU2020 | 0.313 | 0.037 | 0.763 | g__Dongia | Gram-negative, strictly aerobic and heterotrophic bacterium (Liu et al., 2010) |
|  | OTU4132 | 0.327 | 0.026 | 0.661 | g__Granulicella | Strictly aerobic, heterotrophic bacteria (Pankratov and Dedysh, 2010) |
|  | OTU8879 | 0.448 | 0.001 | 0.643 | g__Mucilaginibacter | Aerobic, heterotrophic bacteria capable of degrading pectin, xylan, laminarin and some other polysaccharides (Pankratov et al., 2007) |
|  | OTU1398 | 0.445 | 0.002 | 0.604 | o__Subgroup_2 |  |
|  | OTU6104 | 0.436 | 0.001 | 0.588 | g__Burkholderia-Caballeronia-Paraburkholderia |  |
|  | OTU6871 | 0.472 | 0.001 | 0.511 | o__Acidobacteriales |  |
| **Fungi** | **OTU** | **Indicator value** | **P** | **Relative abundance (%)** | **Taxonomy** | **Description** |
| VS | OTU5425 | 0.333 | 0.024 | 6.312 | g__Pleiochaeta | Species of Pleiochaeta are pathogenic causing leaf spots on legumes (Marin-Felix et al., 2017) |
|  | OTU1969 | 0.665 | 0.001 | 5.767 | g__Fusarium | One of the most important mycotoxigenic fungal genera in food and feed (Thrane, 2014) |
|  | OTU5536 | 0.266 | 0.042 | 2.676 | o__Polyporales | Most of the 1800 or so species of Polyporales cause rot in standing trees and fallen logs (Money, 2016) |
|  | OTU6828 | 0.546 | 0.001 | 2.311 | g__Neocosmospora | Given their importance as plant pathogens, species of Neocosmospora have been used as model organisms in molecular plant pathology (Sandoval-Denis et al., 2019) |
| VLS | OTU1109 | 0.398 | 0.001 | 10.970 | p__Ascomycota |  |
|  | OTU2606 | 0.379 | 0.001 | 8.814 | g__Archaeorhizomyces | Archaeorhizomycetes have saprotrophic potential, yet are omnipresent in roots and rhizosphere soil and show ecosystem and host root habitat specificity (Rosling et al., 2011) |
|  | OTU3955 | 0.336 | 0.047 | 3.459 | s__Orbiliomycetes_sp |  |
|  | OTU4164 | 0.219 | 0.006 | 2.449 | o__Agaricales |  |
| LS | OTU7420 | 0.332 | 0.001 | 19.953 | p__Basidiomycota | The Basidiomycota are important contributors to ecosystem functioning at multiple levels and are the major degraders of different components in wood, including lignin (Taylor et al., 2015) |
|  | OTU6890 | 0.489 | 0.001 | 4.241 | g__Trichoderma | Trichoderma play an important role decomposing plant material |
|  | OTU6204 | 0.337 | 0.028 | 3.988 | p__Ascomycota |  |
|  | OTU6865 | 0.395 | 0.001 | 3.978 | p__Ascomycota |  |
|  | OTU7315 | 0.214 | 0.049 | 3.303 | c__Agaricomycetes |  |
|  | OTU5948 | 0.316 | 0.001 | 2.577 | g__Hygrocybe |  |
|  | OTU3456 | 0.285 | 0.002 | 2.535 | s__Mortierellomycotina |  |
|  | OTU7295 | 0.409 | 0.001 | 2.279 | o__Helotiales |  |

**Table S2** List of bacterial genera in Proteobacteria and Actinobacteriota showing clear expansion in response to species replacement.

| **Phylum** | **Genus** | **Description** |
| --- | --- | --- |
| p__Proteobacteria | g__Acidibacter | Acidophilic, grows at 8-45℃ (optimally at 32–35℃) and at pH 2.5-4.5 (optimally at 3.5) (Falagán and Johnson, 2014) |
| p__Actinobacteriota | g__Angustibacter | Endophytic, grows at 20-30℃ and at pH 6-8 (Tamura, 2020) |
| p__Proteobacteria | g__Arboricoccus | Endophytic, growth occurs at 15-37 ℃ (optimum, 26-30℃), at pH 5.5-8.5 (optimum, pH 6.0-7.0) (Proença et al., 2018) |
| p__Actinobacteriota | g__Arthrobacter | Heterotrophic nitrification, growth occurs at 20-30 ℃ and at neutral to slightly alkaline pH (Gobbetti and Rizzello, 2014) |
| p__Proteobacteria | g__Aureimonas | Carbon and nitrogen cycles, growth occurs at 4-40 ℃ (optimum 25-30℃) (Rathsack et al., 2011) |
| p__Actinobacteriota | g__Catenulispora | Acidophilic, grows well at 11-37℃ (optimum 22-28℃), and pH 4.3-6.8 (optimum 6.0) (Busti et al., 2006) |
| p__Proteobacteria | g__Cellvibrio | Cellulolytic, growth occurs at a temperature range of between 16 and 37℃ |
| p__Actinobacteriota | g__Cryptosporangium | Mesophilic, good growth occurs at 20-25 ℃ (Kämpfer and Glaeser, 2018) |
| p__Proteobacteria | g__Enterobacter | Pathogenic, grow between 6 and 47℃ with a pH range of 4.5-10 (Iversen, 2014) |
| p__Actinobacteriota | g__Friedmanniella | Growth occurs at 15-37℃ (optimum 25℃) at pH 5.5-8.0 (optimum 7.0-7.5) (Schumann and Pukall, 2015) |
| p__Proteobacteria | g__Legionella | Pathogenic, optimal growth temperature for Legionella is 35℃ (Percival and Williams, 2014) |
| p__Proteobacteria | g__Limnobacter | Carbon and nitrogen metabolism, sulfur oxidization, growth occurs between 4 and 38℃ (Spring et al., 2001) |
| p__Proteobacteria | g__Luteibacter | Methionine metabolism, mesophilic (between 5-30 ℃), pH range 6-9 (Johansen et al., 2005) |
| p__Actinobacteriota | g__Marmoricola | Endophytic, mesophilic, grow best at 28-30℃, prefer a neutral to mildly alkaline pH (Evtushenko, 2015) |
| p__Actinobacteriota | g__Microlunatus | Phosphorus metabolism, mesophilic. Good growth occurs at 20-30℃ (Hanada and Nakamura, 2015) |
| p__Actinobacteriota | g__Nocardioides | Play an important role in bioremediation and decompose various pollutants such as alkanes, pyridine, phenols, phenanthrene, etc. (Park et al., 2020) |
| p__Proteobacteria | g__Novosphingobium | Degrade a wide range of xenobiotic compounds, growth occurs at 4-45℃ (optimum 25-30℃) and pH 4-10.5(optimum 6-8) (Kumar et al., 2022) |
| p__Proteobacteria | g__Phreatobacter | Growth occurs at 20-45℃ (optimum at 25-37℃) and at pH 5.5-9.5 (optimum pH 6–8) (Tóth et al., 2014) |
| p__Proteobacteria | g__Pseudorhodoplanes | Has ability to fix nitrogen and produce plant growth promoting regulators, able to grow at pH 5.5-8, at 15-35℃ (Tirandaz et al., 2015) |
| p__Proteobacteria | g__Reyranella | Biodegradation of trace organic compounds, grow well between 30 and 35℃ (Pagnier et al., 2010) |
| p__Proteobacteria | g__Rhizobacter | Rubber-degrading bacterium, growth occurs at 28-30℃, at pH 5.0-9.0 (Goto, 2015) |
| p__Proteobacteria | g__Rhizorhapis | Cause corky root disease, the optimum growth temperature is 28-32℃, the maximum growth temperature is 37℃ (Francis et al., 2014) |
| p__Proteobacteria | g__Rhodovastum | Nitrogen cycle, acidophilic, growth occurs at 20-40℃ (optimum 30-45℃), at pH 5.0-8.5(optimumu pH 6.0-6.5) (Okamura et al., 2009) |

**Table S3** List of bacterial genera in Proteobacteria showing clear decline in response to species replacement.

| **Phylum** | **Genus** | **Description** |
| --- | --- | --- |
| p__Proteobacteria | g__Acidiphilium | Acidophilic, mesophilc, growth occurs at 22-35℃ (optimum, 30℃) and at pH 2.0-5.5 (optimum, pH 3.5) (Okamura et al., 2015) |
| p__Proteobacteria | g__Ahniella | Pollutant degradation, growth occurs at 15-35℃ (optimum 30℃), ranges for growth are pH 6.5-8.0 (optimum pH 7.0) (Tang et al., 2023) |
| p__Proteobacteria | g__Amaricoccus | Degradation of organic compounds, growth occurs at temperatures between 20 and 37℃ and at pH values between 5.5 and 9.0 (Brunhoferova et al., 2022) |
| p__Proteobacteria | g__Aquicella | Plant growth promoting effect in potassium solubilization and plant disease resistance, growth occurs at 30-43℃ and neutral pH (Li et al., 2022) |
| p__Proteobacteria | g__Asticcacaulis | Cetylpyridinium chloride biodegradation, optimal temperature for growth: 25-30℃, optimal pH near neutrality (Poindexter, 2015) |
| p__Proteobacteria | g__Bauldia | Nitrogen-fixing bacteria, optimum growth temperature is 28-30℃ at neutral pH (Staley et al., 2019) |
| p__Proteobacteria | g__Constrictibacter | Degradation of cellulose, grows at 5-30℃ (optimum 25℃) and pH 6.0-8.0 (optimum approximately pH 7.0) (Yamada et al., 2011) |
| p__Proteobacteria | g__Cupriavidus | Herbicide degrading bacteria, optimum temperature is 27℃. Optimum pH is 7.0-8.0. (Makkar and Casida, 1987) |
| p__Proteobacteria | g__Dongia | Nonionic surfactant degradation, growth occurs at 20-40℃ (optimum 30-35℃) and pH 6.0-10.0 (optimum pH 7.0-7.5). (Liu et al., 2010) |
| p__Proteobacteria | g__Dyella | Nitrogen metabolism, optimum pH for growth is 6.5-7.2, optimum temperature for growth is 25-30℃ (Xie and Yokota, 2005) |
| p__Proteobacteria | g__Lysobacter | Control plant diseases, optimum temperature is 28℃, optimum pH for growth is 7.0-7.5 (Park et al., 2008) |
| p__Proteobacteria | g__Nevskia | Mn oxidizers, optimum growth temperature is about 30℃, optimum pH for growth is 6.0-6.5; (Leandro et al., 2012) |
| p__Proteobacteria | g__Nordella | Nitrogen cycle, grow well at 25 and 30℃ (La Scola et al., 2004) |
| p__Proteobacteria | g__Pedomicrobium | Mn oxidizers, grow well at 20-30℃ and in the pH range of 7-9 (Hirsch, 2015) |
| p__Proteobacteria | g__Phenylobacterium | Degrading xenobiotic compounds, optimum growth at 28-30℃ and at pH 6.8-7.0. (Lingens et al., 1985) |
| p__Proteobacteria | g__Phyllobacterium | Nitrogen fixation, optimal temperature for growth, 28-34℃. (Mergaert and Swings, 2015) |
| p__Proteobacteria | g__Rhodomicrobium | Psychrotolerant, nitrogen Fixation, capable of growth at low temperatures (10-15℃) (Ramana et al., 2013) |
| p__Proteobacteria | g__Rickettsia | Pathogenic, the optimal growth temperature is 32-34℃. (Liu, 2015) |
| p__Proteobacteria | g__Rivibacter | Heterotrophic denitrifiers, optimum growth at 25-28℃ and at pH 8.4-8.9 (Stackebrandt et al., 2009) |
| p__Proteobacteria | g__Sphingoaurantiacus | Growth occurred at 4-37℃ (optimum 28-30℃) and at pH 6.0-10.0 (optimum pH 9.0). (Kim et al., 2016) |

**Table S4** List of fungal genera in Ascomycota showing clear decline in response to species replacement.

| **Phylum** | **Genus** | **Description** |
| --- | --- | --- |
| p__Ascomycota | g__Albifimbria | Soil inhabitants and saprophytes of decaying plant material (Matic et al., 2019) |
| p__Ascomycota | g__Alfaria | Plant pathogenic fungi (Crous et al., 2014) |
| p__Ascomycota | g__Alternaria | Plant parasites, saprobes, and endophytes (Lawrence et al., 2015) |
| p__Ascomycota | g__Apiospora | Endophytic fungus, worldwide (tropical, subtropical, temperate, and cold regions) (Pintos and Alvarado, 2021) |
| p__Ascomycota | g__Aureobasidium | Endophytic fungi (Tsujisaka and Mitsuhashi, 1993) |
| p__Ascomycota | g__Auxarthron | Isolated from anthropized soil (Sarrocco et al., 2015) |
| p__Ascomycota | g__Chaetomium | Endophytic fungi (Tsujisaka and Mitsuhashi, 1993) |
| p__Ascomycota | g__Chaetosphaeria | Saprobic fungi (Fernandez et al., 2006) |
| p__Ascomycota | g__Cladophialophora | Plant saprophytes and endophytes (Davey and Currah, 2007) |
| p__Ascomycota | g__Clonostachys | Nematophagous fungi (Soares et al., 2020) |
| p__Ascomycota | g__Colletotrichum | Endophytic fungi (Tsujisaka and Mitsuhashi, 1993) |
| p__Ascomycota | g__Coniochaeta | Endophytic fungi (Xie et al., 2015) |
| p__Ascomycota | g__Conlarium | Endophytic fungi (Xie et al., 2019) |
| p__Ascomycota | g__Devriesia | Originate from plants, soil, air and rocks |
| p__Ascomycota | g__Endophragmiella | Saprophytic fungus |
| p__Ascomycota | g__Epicoccum | Foliicolous fungi |
| p__Ascomycota | g__Forliomyces | Saprophytic fungus (Phukhamsakda et al., 2016) |
| p__Ascomycota | g__Fusariella | Saprophytic fungus |
| p__Ascomycota | g__Fusidium | Endophytic fungi |
| p__Ascomycota | g__Gibberella | Plant pathogens |
| p__Ascomycota | g__Knufia | Rock‑inhabiting fungus (Tesei et al., 2021) |
| p__Ascomycota | g__Lecanicillium | Entomopathogenic fungi (Ishidoh et al., 2014) |
| p__Ascomycota | g__Leohumicola | Endophytic heat-resistant fungi (Hambleton et al., 2005) |
| p__Ascomycota | g__Leptodiscella | Isolated from leaf litter and soil |
| p__Ascomycota | g__Metapochonia | Parasitic on nematode eggs or cysts (Kondo et al., 2020) |
| p__Ascomycota | g__Microdiplodia | Endophytic fungi |
| p__Ascomycota | g__Montagnula | Saprophytic fungus (Tennakoon et al., 2016) |
| p__Ascomycota | g__Myxospora | Saprophytic fungus |
| p__Ascomycota | g__Ochroconis | Human pathogen |
| p__Ascomycota | g__Ophiocordyceps | Insect host species (Wang and Yao, 2011) |
| p__Ascomycota | g__Paraphoma | Saprophytic, soil-borne pathogens (Cao and Li, 2022) |
| p__Ascomycota | g__Penicillium | Many of which are soil inhabitants, and give us penicillin (Houbraken et al., 2014) |
| p__Ascomycota | g__Pleotrichocladium | Root symbiotic fungi (Xu et al., 2022) |
| p__Ascomycota | g__Preussia | Endophytic fungi |
| p__Ascomycota | g__Sagenomella | Soil-borne ascomycetes (Endo et al., 1998) |
| p__Ascomycota | g__Scytalidium | Human pathogen |
| p__Ascomycota | g__Sepedonium | Saprophytic fungus |
| p__Ascomycota | g__Setophaeosphaeria | Plant pathogens (Phookamsak et al., 2014) |
| p__Ascomycota | g__Sirastachys | Saprophytic fungus |
| p__Ascomycota | g__Sphaerulina | Plant-pathogenic fungi |
| p__Ascomycota | g__Stagonospora | Plant-pathogenic fungi |
| p__Ascomycota | g__Striaticonidium |  |
| p__Ascomycota | g__Stylonectria |  |
| p__Ascomycota | g__Talaromyces | Soil inhabitants, some of them are heat resistant (Pitt, 2014) |
| p__Ascomycota | g__Tetracladium | Saprophytic fungus, root endophytes (Lazar et al., 2022) |
| p__Ascomycota | g__Thelonectria | Saprophytic fungus (Salgado-Salazar et al., 2016) |
| p__Ascomycota | g__Trichoderma | Soil-borne ascomycetes |

**Table S5** List of fungal genera in Ascomycota showing clear expansion in response to species replacement.

| **Phylum** | **Genus** | **Description** |
| --- | --- | --- |
| p__Ascomycota | g__Arthrocatena | Rock-inhabiting fungi (Egidi et al., 2014) |
| p__Ascomycota | g__Biappendiculispora | Saprobic on woody and herbaceous substrates (Tanaka and Harada, 2003) |
| p__Ascomycota | g__Bradymyces | Rock-inhabiting fungi (Hubka et al., 2014) |
| p__Ascomycota | g__Cyphellophora | Growth occurred at 21-36℃ (optimum 21-27℃), plant-inhabiting fungus (Feng et al., 2014) |
| p__Ascomycota | g__Dactylellina | Nematode-trapping fungus |
| p__Ascomycota | g__Dictyosporium | Saprophytic fungus (Yang et al., 2018) |
| p__Ascomycota | g__Dothiorella | Endophytic pathogenic fungus (You et al., 2017) |
| p__Ascomycota | g__Fusicolla | Endophytic pathogenic fungus (Li et al., 2021) |
| p__Ascomycota | g__Gonytrichum | Mutualistic fungi (Purwati et al., 2019) |
| p__Ascomycota | g__Metarhizium | Entomogenous fungi (Sung et al., 2007) |
| p__Ascomycota | g__Neocosmospora | Contains saprobes, plant endophytes and pathogens as well as animal pathogens (Sandoval-Denis et al., 2019) |
| p__Ascomycota | g__Neophaeococcomyces | Rock-inhabiting fungi (Crous et al., 2021) |
| p__Ascomycota | g__Phialophora | Endophytic fungus (Cheng et al., 2021) |
| p__Ascomycota | g__Plenodomus | Generally saprophytes but also parasitic on herbaceous or woody plants (Naing et al., 2020) |
| p__Ascomycota | g__Pochonia | Nematophagous endophytic fungus (Gouveia et al., 2022) |
| p__Ascomycota | g__Pseudogymnoascus | Psychrophilic pathogenic fungus (Shi et al., 2021) |
| p__Ascomycota | g__Purpureocillium | Nematode-trapping fungi (Senthilkumar et al., 2020) |
| p__Ascomycota | g__Stenella | Foliicolous fungi |
| p__Ascomycota | g__Trematosphaeria | Saprobic or hemibiotrophic on terrestrial plants (Zhou et al., 2014) |

**References**

Beeckmans, S., and Xie, J.P. (2015). "Glyoxylate Cycle" in *Reference Module in Biomedical Science*s. Eds, M. Caplan (Elsevier Ltd) 1-23.

Brunhoferova, H., Venditti, S., Laczny, C.C., Lebrun, L., and Hansen, J. (2022). Bioremediation of 27 micropollutants by symbiotic microorganisms of wetland macrophytes. *Sustainability* 14(7). doi: 10.3390/su14073944.

Busti, E., Cavaletti, L., Monciardini, P., Schumann, P., Rohde, M., Sosio, M., et al. (2006). *Catenulispora acidiphila* gen. nov., sp. nov., a novel, mycelium-forming actinomycete, and proposal of *Catenulisporaceae* fam. nov. *Int*. *J*. *Syst*. *Evol*. *Microbiol*. 56(Pt 8), 1741-1746. doi: 10.1099/ijs.0.63858-0.

Cao, S., and Li, Y.Z. (2022). Growth, sporulation, conidial germination and lethal temperature of *Paraphoma* *radicina*, a fungal pathogen of Alfalfa (*Medicago* *sativa*) root rot. *Agriculture-Basel* 12(9). doi: 10.3390/agriculture12091501.

Cheng, M.-J., Wu, M.-D., Chen, C.-Y., Hsieh, S.-Y., and Chen, J.-J. (2021). Metabolite from the fungus of *Phialophora* *lagerbergii*. *Chem*. *Nat*. *Compd*. 57(6), 1032-1034. doi: 10.1007/s10600-021-03543-3.

Crous, P.W., Cowan, D.A., Maggs-Kolling, G., Yilmaz, N., Thangavel, R., Wingfield, M.J., et al. (2021). Fungal Planet description sheets: 1182-1283. *Persoonia* 46, 313-528. doi: 10.3767/persoonia.2021.46.11.

Crous, P.W., Shivas, R.G., Quaedvlieg, W., van der Bank, M., Zhang, Y., Summerell, B.A., et al. (2014). Fungal Planet description sheets: 214-280. *Persoonia* 32, 184-306. doi: 10.3767/003158514x682395.

Davey, M.L., and Currah, R.S. (2007). A new species of *Cladophialophora* (hyphomycetes) from boreal and montane bryophytes. *Mycol*. *Res*. 111, 106-116. doi: 10.1016/j.mycres.2006.10.004.

Egidi, E., de Hoog, G.S., Isola, D., Onofri, S., Quaedvlieg, W., de Vries, M., et al. (2014). Phylogeny and taxonomy of meristematic rock-inhabiting black fungi in the Dothideomycetes based on multi-locus phylogenies. *Fungal Divers*. 65(1), 127-165. doi: 10.1007/s13225-013-0277-y.

Endo, M., Thanh, N.T., Yokota, A., Gams, W., and Sugiyama, S. (1998). Phylogenetic analysis of *Sagenomella* and relatives based on nuclear 18S ribosomal RNA gene and determination of ubiquinone system. *Biseibutsu Bunrui Kenkyukai Puroguramu oyobi Shoroku* 18, 35-36.

Evtushenko, L. (2015). "Marmoricola" in *Bergey's Manual of Systematics of Archaea and Bacteria*. Eds, M.E. Trujillo, S. Dedysh, P. DeVos, B. Hedlund, P. Kämpfer, F.A. Rainey, and W.B. Whitman (New York: John Wiley & Sons, Inc), 1–27.

Falagán, C., and Johnson, D.B. (2014). *Acidibacter ferrireducens* gen. nov., sp. nov.: an acidophilic ferric iron-reducing gammaproteobacterium. *Extremophiles* 18(6), 1067-1073. doi: 10.1007/s00792-014-0684-3.

Feng, P., Lu, Q., Najafzadeh, M.J., Gerrits van den Ende, A.H.G., Sun, J., Li, R., et al. (2014). *Cyphellophora* and its relatives in *Phialophora*: biodiversity and possible role in human infection. *Fungal Divers*. 65(1), 17-45. doi: 10.1007/s13225-012-0194-5.

Fernandez, F.A., Miller, A.N., Huhndorf, S.M., Lutzoni, F.M., and Zoller, S. (2006). Systematics of the genus *Chaetosphaeria* and its allied genera: morphological and phylogenetic diversity in north temperate and neotropical taxa. *Mycologia* 98(1), 121-130. doi: 10.3852/mycologia.98.1.121.

Francis, I.M., Jochimsen, K.N., De Vos, P., and van Bruggen, A.H.C. (2014). Reclassification of rhizosphere bacteria including strains causing corky root of lettuce and proposal of *Rhizorhapis suberifaciens* gen. nov., comb. nov., *Sphingobium mellinum* sp. nov., *Sphingobium xanthum* sp. nov. and *Rhizorhabdus argentea* gen. nov., sp. nov. *Int*. *J*. *Syst*. *Evol*. *Microbiol*. 64(Pt_4), 1340-1350. doi: 10.1099/ijs.0.058909-0.

García-Fraile, P., Benada, O., Cajthaml, T., Baldrian, P., and Lladó, S. (2016). *Terracidiphilus* *gabretensis* gen. nov., sp. nov., an abundant and active forest soil acidobacterium important in organic matter transformation. *Appl*. *Environ*. *Microbiol*. 82(2), 560-569. doi: 10.1128/aem.03353-15.

Gobbetti, M., and Rizzello, C.G. (2014). "Arthrobacter" in *Encyclopedia of Food Microbiology (Second Edition),* Eds. C.A. Batt and M.L. Tortorello (Oxford: Academic Press), 69-76.

Goto, M. (2015). "Rhizobacter" in *Bergey's Manual of Systematics of Archaea and Bacteria*. Eds, M.E. Trujillo, S. Dedysh, P. DeVos, B. Hedlund, P. Kämpfer, F.A. Rainey, and W.B. Whitman (New York: John Wiley & Sons, Inc), 1-5.

Gouveia, A.D., Monteiro, T.S.A., Luiz, P.H.D., Balbino, H.M., de Magalhaes, F.C., de Moura, V.A.S., et al. (2022). The nematophagous root endophyte *Pochonia* *chlamydosporia* alters tomato metabolome. *Rhizosphere* 22. doi: 10.1016/ j.rhisph.2022.100531.

Hambleton, S., Nickerson, N.L., and Seifert, K.A. (2005). *Leohumicola*, a new genus of heat-resistant hyphomycetes. *Stud*. *Mycol*. (53), 29-52. doi: 10.3114/ sim.53.1.29.

Hanada, S., and Nakamura, K. (2015). "Microlunatus" in *Bergey's Manual of Systematics of Archaea and Bacteria*. Eds, M.E. Trujillo, S. Dedysh, P. DeVos, B. Hedlund, P. Kämpfer, F.A. Rainey, and W.B. Whitman (New York: John Wiley & Sons, Inc), 1-7.

Hirsch, P. (2015). "Pedomicrobium" in *Bergey's Manual of Systematics of Archaea and Bacteria*. Eds, M.E. Trujillo, S. Dedysh, P. DeVos, B. Hedlund, P. Kämpfer, F.A. Rainey, and W.B. Whitman (New York: John Wiley & Sons, Inc), 1-18.

Houbraken, J., de Vries, R. P., & Samson, R. A. (2014). Modern taxonomy of biotechnologically important *Aspergillus* and *Penicillium* species. *Adv*. *Appl*. *Microbiol*. 86, 199-249. doi: 10.1016/B978-0-12-800262-9.00004-4.

Huber, K.J., and Overmann, J. (2018). *Vicinamibacteraceae* fam. nov., the first described family within the subdivision 6 *Acidobacteria*. *Int*. *J*. *Syst*. *Evol*. *Microbiol*. 68(7), 2331-2334. doi: 10.1099/ijsem.0.002841.

Hubka, V., Réblová, M., Řehulka, J., Selbmann, L., Isola, D., de Hoog, S.G., et al. (2014). *Bradymyces* gen. nov. (Chaetothyriales, Trichomeriaceae), a new ascomycete genus accommodating poorly differentiated melanized fungi. *Antonie van Leeuwenhoek* 106(5), 979-992. doi: 10.1007/s10482-014-0267-4.

Ishidoh, K., Kinoshita, H., Ihara, F., and Nihira, T. (2014). Efficient and versatile transformation systems in entomopathogenic fungus *Lecanicillium* species. *Curr*. *Genet*. 60(2), 99-108. doi: 10.1007/s00294-013-0399-5.

Iversen, C. (2014). "Electrical Techniques: Enterobacter" in *Encyclopedia of food microbiology*. (Elsevier Ltd), 653-658.

Johansen, J.E., Binnerup, S.J., Kroer, N., and Mølbak, L. (2005). *Luteibacter rhizovicinus* gen. nov., sp. nov., a yellow-pigmented gammaproteobacterium isolated from the rhizosphere of barley (*Hordeum vulgare* L.). *Int*. *J*. *Syst*. *Evol*. *Microbiol*. 55(6), 2285-2291. doi: 10.1099/ijs.0.63497-0.

Kämpfer, P., and Glaeser, S. (2018). "Cryptosporangium" in *Bergey's Manual of Systematics of Archaea and Bacteria*. Eds, M.E. Trujillo, S. Dedysh, P. DeVos, B. Hedlund, P. Kämpfer, F.A. Rainey, and W.B. Whitman (New York: John Wiley & Sons, Inc), 1-9.

Kim, M., Kang, O., Zhang, Y., Ren, L., Chang, X., Jiang, F., et al. (2016). *Sphingoaurantiacus polygranulatus* gen. nov., sp. nov., isolated from high-Arctic tundra soil, and emended descriptions of the genera *Sandarakinorhabdus*, *Polymorphobacter* and *Rhizorhabdus* and the species *Sandarakinorhabdus limnophila*, *Rhizorhabdus* argentea and *Sphingomonas wittichii*. *Int*. *J*. *Syst*. *Evol*. *Microbiol*. 66(1), 91-100. doi: 10.1099/ijsem.0.000677.

Kondo, N., Tokiwa, T., Sato, K., Omura, S., and Nonaka, K. (2020). *Metapochonia* *hahajimaensis* (Clavicipitaceae, Hypocreales), a new species from soil in Hahajima Island, Tokyo, Japan. *Mycoscience* 61(6), 337-341. doi: 10.1016/ j.myc.2020.06.001.

Kumar, R., Kumari, S., Anil Kumar, P., and Lal, R. (2022). "Novosphingobium" in *Bergey's Manual of Systematics of Archaea and Bacteria*. Eds. M.E. Trujillo, S. Dedysh, P. DeVos, B. Hedlund, P. Kämpfer, F.A. Rainey, and W.B. Whitman (New York: John Wiley & Sons, Inc), 1-24.

Kuramae, E.E., and de Assis Costa, O.Y. (2019). "Acidobacteria" in *Encyclopedia of Microbiology (Fourth Edition)*. Eds. T.M. Schmidt. (Oxford: Academic Press), 1-8.

La Scola, B., Barrassi, L., and Raoult, D. (2004). A novel alpha-Proteobacterium, *Nordella oligomobilis* gen. nov., sp. nov., isolated by using amoebal co-cultures. *Res*. *Microbiol*. 155(1), 47-51. doi: 10.1016/j.resmic.2003.09.012.

Lawrence, D.P., Rotondo, F., and Gannibal, P.B. (2015). Biodiversity and taxonomy of the pleomorphic genus *Alternaria*. *Mycol*. *Prog*. 15(1), 3. doi: 10.1007/s11557-015-1144-x.

Lazar, A., Mushinski, R.M., and Bending, G.D. (2022). Landscape scale ecology of *Tetracladium* spp. fungal root endophytes. *Environ*. *Microbiome* 17(1). doi: 10.1186/s40793-022-00431-3.

Leandro, T., França, L., Nobre, M.F., Schumann, P., Rosselló-Móra, R., and da Costa, M.S. (2012). *Nevskia aquatilis* sp. nov. and *Nevskia persephonica* sp. nov., isolated from a mineral water aquifer and the emended description of the genus *Nevskia*. *Syst*. *Appl*. *Microbiol*. 35(5), 297-301. doi: 10.1016/j.syapm.2012.05. 001.

Lee, H.J., Lee, S.H., Lee, S.S., Lee, J.S., Kim, Y., Kim, S.C., et al. (2014). *Ramlibacter solisilvae* sp. nov., isolated from forest soil, and emended description of the genus *Ramlibacter*. *Int*. *J*. *Syst*. *Evol*. *Microbiol*. 64(Pt 4), 1317-1322. doi: 10.1099/ijs.0.058396-0.

Li, W.Z., Long, Y.H., Mo, F.X., Shu, R., Yin, X.H., Wu, X.M., et al. (2021). Antifungal activity and biocontrol mechanism of *Fusicolla violacea* J-1 against soft rot in Kiwifruit caused by *Alternaria alternata*. *J*. *Fungi* 7(11). doi: 10.3390/ jof7110937.

Li, Y.Z., Li, T., Wang, Z.T., Wang, S.N., Qin, X.L., and Liao, Y.C. (2022). Plastic film mulch changes the microbial community in maize root-associated compartments. *Plant Soil* 470(1-2), 5-20. doi: 10.1007/s11104-021-05060-2.

Lingens, F., Blecher, R., Blecher, H., Blobel, F., Eberspächer, J., Fröhner, C., et al. (1985). *Phenylobacterium immobile* gen. nov., sp. nov., a gram-negative bacterium that degrades the herbicide chloridazon. *Int*. *J*. *Syst*. *Evol*. *Microbiol*. 35, 26-39.

Liu, D. (2015). "Rickettsia" in *Molecular Medical Microbiology (Second Edition)*.Eds, Y.-W. Tang, M. Sussman, D. Liu, I. Poxton, and J. Schwartzman (Boston: Academic Press), 2043-2056.

Liu, Y., Jin, J.H., Liu, Y.H., Zhou, Y.G., and Liu, Z.P. (2010). *Dongia mobilis* gen. nov., sp. nov., a new member of the family *Rhodospirillaceae* isolated from a sequencing batch reactor for treatment of malachite green effluent. *Int*. *J*. *Syst*. *Evol*. *Microbiol*. 60(Pt 12), 2780-2785. doi: 10.1099/ijs.0.020347-0.

Makkar, N.S., and Casida, L.E. (1987). *Cupriavidus necator* gen. nov., sp. nov.; a nonobligate bacterial predator of bacteria in soil. *Int*. *J*. *Syst*. *Evol*. *Microbiol*. 37(4), 323-326. doi: 10.1099/00207713-37-4-323.

Marin-Felix, Y., Groenewald, J.Z., Cai, L., Chen, Q., Marincowitz, S., Barnes, I., et al. (2017). Genera of phytopathogenic fungi: Gophy 1. *Stud*. *Mycol*. 86, 99-216. doi: 10.1016/j.simyco.2017.04.002.

Matic, S., Gilardi, G., Gullino, M.L., and Garibaldi, A. (2019). Emergence of leaf spot disease on leafy vegetable and ornamental crops caused by *Paramyrothecium* and *Albifimbria* species. *Phytopathology* 109(6), 1053-1061. doi: 10.1094/phyto-10-18-0396-r.

Mergaert, J., and Swings, J. (2015). "Phyllobacterium" in *Bergey's Manual of Systematics of Archaea and Bacteria*. Eds, M.E. Trujillo, S. Dedysh, P. DeVos, B. Hedlund, P. Kämpfer, F.A. Rainey, and W.B. Whitman (New York: John Wiley & Sons, Inc), 1-7.

Money, N.P. (2016). "Fungal Diversity" in *The Fungi (Third Edition),* Eds, S.C. Watkinson, L. Boddy, and N.P. Money (Boston: Academic Press), 1-36.

Naing, M.T., Kallol, D., In-Kyu, K., Seung-Yeol, L., and Hee-Young, J. (2020). Morphological and phylogeny of *Plenodomus* *sinensis* and *P*. *collinsoniae*, two unreported species isolated from soil in Korea. *The Korean Journal of Mycology* 48(3), 187-195. doi: 10.4489/kjm.20200020.

Okamura, K., Hisada, T., Kanbe, T., and Hiraishi, A. (2009). *Rhodovastum atsumiense* gen. nov., sp. nov., a phototrophic alphaproteobacterium isolated from paddy soil. *J*. *Gen*. *Appl*. *Microbiol*. 55(1), 43-50. doi: 10.2323/jgam.55.43.

Okamura, K., Kawai, A., Wakao, N., Yamada, T., and Hiraishi, A. (2015). *Acidiphilium iwatense* sp. nov., isolated from an acid mine drainage treatment plant, and emendation of the genus *Acidiphilium*. *Int*. *J*. *Syst*. *Evol*. *Microbiol*. 65(Pt 1), 42-48. doi: 10.1099/ijs.0.065052-0.

Pagnier, I., Raoult, D., and la Scola, B. (2010). Isolation and characterization of *Reyranella massiliensis* gen. nov., sp. nov. from freshwater samples by using an amoeba co-culture procedure. *Int*. *J*. *Syst*. *Evol*. *Microbiol*. 61, 2151-2154. doi: 10.1099/ijs.0.025775-0.

Pankratov, T.A., and Dedysh, S.N. (2010). *Granulicella paludicola* gen. nov., sp. nov., *Granulicella pectinivorans* sp. nov., *Granulicella aggregans* sp. nov. and *Granulicella rosea* sp. nov., acidophilic, polymer-degrading acidobacteria from *Sphagnum* peat bogs. *Int*. *J*. *Syst*. *Evol*. *Microbiol*. 60(Pt 12), 2951-2959. doi: 10.1099/ijs.0.021824-0.

Pankratov, T.A., Tindall, B.J., Liesack, W., and Dedysh, S.N. (2007). *Mucilaginibacter* *paludis* gen. nov., sp. nov. and *Mucilaginibacter* *gracilis* sp. nov., pectin-, xylan- and laminarin-degrading members of the family *Sphingobacteriaceae* from acidic *Sphagnum* peat bog. *Int*. *J*. *Syst*. *Evol*. *Microbiol*. 57(Pt 10), 2349-2354. doi: 10.1099/ijs.0.65100-0.

Park, J.H., Kim, R., Aslam, Z., Jeon, C.O., and Chung, Y.R. (2008). *Lysobacter* *capsici* sp. nov., with antimicrobial activity, isolated from the rhizosphere of pepper, and emended description of the genus *Lysobacter*. *Int*. *J*. *Syst*. *Evol*. *Microbiol*. 58(Pt 2), 387-392. doi: 10.1099/ijs.0.65290-0.

Park, Y., Liu, Q.Z., Maeng, S., Choi, W.J., Chang, Y., and Im, W.T. (2020). *Nocardioides* *convexus* sp. nov. and *Nocardioides* *anomalus* sp. nov., isolated from soil and mineral water. *Int*. *J*. *Syst*. *Evol*. *Microbiol*. 70(12), 6402-6407. doi: 10.1099/ijsem.0.004547.

Percival, S.L., and Williams, D.W. (2014). "Legionella" in *Microbiology of Waterborne Diseases (Second Edition)*. Eds. S.L. Percival, M.V. Yates, D.W. Williams, R.M. Chalmers, and N.F. Gray (London: Academic Press), 155-175.

Phookamsak, R., Liu, J.K., McKenzie, E.H.C., Manamgoda, D.S., Ariyawansa, H., Thambugala, K.M., et al. (2014). Revision of *Phaeosphaeriaceae*. *Fungal Divers*. 68(1), 159-238. doi: 10.1007/s13225-014-0308-3.

Phukhamsakda, C., Ariyawansa, H.A., Phillips, A.J.L., Wanasinghe, D.N., Bhat, D.J., McKenzie, E.H.C., et al. (2016). Additions to *Sporormiaceae*: Introducing two novel genera, *Sparticola* and *Forliomyces*, from Spartium. *Cryptogamie Mycol*. 37(1), 75-97. doi: 10.7872/crym/v37.iss1.2016.75.

Pintos, A., and Alvarado, P. (2021). Phylogenetic delimitation of *Apiospora* and *Arthrinium*. *Fungal systematics and evolution* 7, 197-221. doi: 10.3114/ fuse.2021.07.10.

Pitt, J.I. (2014). "Penicillium: Penicillium and Talaromyces: Introduction" in *Encyclopedia of Food Microbiology (Second Edition),* Eds. C.A. Batt and M.L. Tortorello (Oxford: Academic Press), 6-13.

Poindexter, J.S. (2015). "Asticcacaulis" in *Bergey's Manual of Systematics of Archaea and Bacteria*. Eds, M.E. Trujillo, S. Dedysh, P. DeVos, B. Hedlund, P. Kämpfer, F.A. Rainey, and W.B. Whitman (New York: John Wiley & Sons, Inc), 1-14.

Proença, D.N., Whitman, W.B., Varghese, N., Shapiro, N., Woyke, T., Kyrpides, N.C., et al. (2018). *Arboriscoccus pini* gen. nov., sp. nov., an endophyte from a pine tree of the class *Alphaproteobacteria*, emended description of *Geminicoccus* *roseus*, and proposal of *Geminicoccaceae* fam. nov. *Syst*. *Appl*. *Microbiol*. 41(2), 94-100. doi: 10.1016/j.syapm.2017.11.006.

Purwati, E S; Dwiputranto, U; Ekowati, N; Ratnaningtyas, N I (2020). Rhizospheric soil fungi from several food crops in Purwokerto. IOP Conference Series: Earth and Environmental Science, 550, 012018. doi:10.1088/1755-1315/550/1/ 012018.

Ramana, V.V., Raj, P.S., Tushar, L., Sasikala, C., and Ramana, C.V. (2013). *Rhodomicrobium* *udaipurense* sp nov., a psychrotolerant, phototrophic alphaproteobacterium isolated from a freshwater stream. *Int*. *J*. *Syst*. *Evol*. *Microbiol*. 63, 2684-2689. doi: 10.1099/ijs.0.046409-0.

Rathsack, K., Reitner, J., Stackebrandt, E., and Tindall, B.J. (2011). Reclassification of *Aurantimonas* *altamirensis* (Jurado et al. 2006), *Aurantimonas ureilytica* (Weon et al. 2007) and *Aurantimonas frigidaquae* (Kim et al. 2008) as members of a new genus, *Aureimonas* gen. nov., as *Aureimonas altamirensis* gen. nov., comb. nov., *Aureimonas ureilytica* comb. nov. and *Aureimonas frigidaquae* comb. nov., and emended descriptions of the genera *Aurantimonas* and *Fulvimarina*. *Int*. *J*. *Syst*. *Evol*. *Microbiol*. 61(Pt 11), 2722-2728. doi: 10.1099/ijs.0.027029-0.

Rosling, A., Cox, F., Cruz-Martinez, K., Ihrmark, K., Grelet, G.-A., Lindahl, B.D., et al. (2011). Archaeorhizomycetes: Unearthing an ancient class of ubiquitous soil fungi. *Science* 333(6044), 876-879. doi: doi:10.1126/science.1206958.

Salgado-Salazar, C., Rossman, A.Y., and Chaverri, P. (2016). The genus *Thelonectria* (*Nectriaceae*, *Hypocreales*, *Ascomycota*) and closely related species with cylindrocarpon-like asexual states. *Fungal Divers*. 80(1), 411-455. doi: 10.1007/s13225-016-0365-x.

Sandoval-Denis, M., Lombard, L., and Crous, P.W. (2019). Back to the roots: a reappraisal of *Neocosmospora*. *Persoonia* 43, 90-185. doi: 10.3767/persoonia. 2019.43.04.

Sarrocco, S., Diquattro, S., Baroncelli, R., Cimmino, A., Evidente, A., Vannacci, G., et al. (2015). A polyphasic contribution to the knowledge of *Auxarthron* (Onygenaceae). *Mycol*. *Prog*. 14(11). doi: 10.1007/s11557-015-1128-x.

Schumann, P., and Pukall, R. (2015). "Friedmanniella" in *Bergey's Manual of Systematics of Archaea and Bacteria*. Eds, M.E. Trujillo, S. Dedysh, P. DeVos, B. Hedlund, P. Kämpfer, F.A. Rainey, and W.B. Whitman (New York: John Wiley & Sons, Inc),1-10.

Senthilkumar, M., Anandham, R., and Krishnamoorthy, R. (2020). "Paecilomyces" in *Beneficial Microbes in Agro-Ecology*. Eds, N. Amaresan, M. Senthil Kumar, K. Annapurna, K. Kumar, and A. Sankaranarayanan (Elsevier Inc), 793-808.

Shi, T., Yu, Y.Y., Dai, J.J., Zhang, Y.T., Hu, W.P., Zheng, L., et al. (2021). New Polyketides from the Antarctic fungus *Pseudogymnoascus* sp. HSX2#-11. *Mar*. *Drugs* 19(3). doi: 10.3390/md19030168.

Soares, F.E.d.F., Gôlo, P.S., and Fernandes, É.K.K. (2020). "Nematophagous and entomopathogenic fungi: new insights into the beneficial fungus-plant interaction" in *Molecular Aspects of Plant Beneficial Microbes in Agriculture*. Eds, V. Sharma, R. Salwan, and L.K.T. Al-Ani (Elsevier Inc), 295-304.

Spring, S., Kämpfer, P., and Schleifer, K.H. (2001). *Limnobacter* *thiooxidans* gen. nov., sp. nov., a novel thiosulfate-oxidizing bacterium isolated from freshwater lake sediment. *Int*. *J*. *Syst*. *Evol*. *Microbiol*. 51(Pt 4), 1463-1470. doi: 10.1099/ 00207713-51-4-1463.

Stackebrandt, E., Verbarg, S., Frühling, A., Busse, H.-J., and Tindall, B. (2009). Dissection of the genus *Methylibium*: Reclassification of *Methylibium fulvum* as Rhizobacter fulvus comb. nov., *Methylibium aquaticum* as *Piscinibacter aquaticus* gen. nov., comb. nov. and *Methylibium subsaxonicum* as *Rivibacter subsaxonicus* gen. nov., comb. nov. and emended descriptions of the genera *Rhizobacter* and *Methylibium*. *Int*. *J*. *Syst*. *Evol*. *Microbiol*. 59, 2552-2560. doi: 10.1099/ijs.0.008383-0.

Staley, J.T., Vasilyeva, L., and Yee, B. (2019). "Bauldia" in *Bergey's Manual of Systematics of Archaea and Bacteria*. Eds, M.E. Trujillo, S. Dedysh, P. DeVos, B. Hedlund, P. Kämpfer, F.A. Rainey and W.B. Whitman (New York: John Wiley & Sons, Inc), 1-5.

Stone, B.W., Li, J., Koch, B.J., Blazewicz, S.J., Dijkstra, P., Hayer, M., et al. (2021). Nutrients cause consolidation of soil carbon flux to small proportion of bacterial community. *Nat*. *Commun*. 12(1), 3381. doi: 10.1038/s41467-021-23676-x.

Sung, G.-H., Hywel-Jones, N.L., Sung, J.-M., Luangsa-ard, J.J., Shrestha, B., and Spatafora, J.W. (2007). Phylogenetic classification of Cordyceps and the clavicipitaceous fungi. *Stud*. *Mycol*. 57, 5-59. doi: 10.3114/sim.2007.57.01.

Tamura, T. (2020). "Angustibacter" in *Bergey's Manual of Systematics of Archaea and Bacteria*. Eds, M.E. Trujillo, S. Dedysh, P. DeVos, B. Hedlund, P. Kämpfer, F.A. Rainey, and W.B. Whitman (New York: John Wiley & Sons, Inc), 1-7.

Tanaka, K., and Harada, Y. (2003). Pleosporales in Japan (1): the genus *Lophiostoma*. *Mycoscience* 44(2), 85-96. doi: 10.1007/S10267-002-0085-9.

Tang, Y.Y., Li, T., Xu, Y.J., Ren, H.Q., and Huang, H. (2023). Effects of electrical stimulation on purification of secondary effluent containing chlorophenols by denitrification biofilter. *Environ*. *Res*. 216. doi: 10.1016/j.envres.2022.114535.

Taylor, T.N., Krings, M., and Taylor, E.L. (2015). "Basidiomycota" in *Fossil Fungi*. Eds. T.N. Taylor, M. Krings, and E.L. Taylor (San Diego: Academic Press), 173-199.

Tennakoon, D.S., Hyde, K.D., Wanasinghe, D.N., Bahkali, A.H., Camporesi, E., Khan, S., et al. (2016). Taxonomy and phylogenetic appraisal of *Montagnula jonesii* sp nov (Didymosphaeriaceae, Pleosporales). *Mycosphere* 7(9), 1346-1356. doi: 10.5943/mycosphere/7/9/8.

Tesei, D., Chiang, A.J., Kalkum, M., Stajich, J.E., Mohan, G.B.M., Sterflinger, K., et al. (2021). Effects of simulated microgravity on the proteome and secretome of the polyextremotolerant black fungus *Knufia chersonesos*. *Front*. *Genet*. 12. doi: 10.3389/fgene.2021.638708.

Thrane, U. (2014). "Fusarium" in *Encyclopedia of Food Microbiology (Second Edition)*. Eds. C.A. Batt and M.L. Tortorello (Oxford: Academic Press), 76-81.

Tirandaz, H., Dastgheib, S.M.M., Amoozegar, M.A., Shavandi, M., de la Haba, R.R., and Ventosa, A. (2015). *Pseudorhodoplanes sinuspersici* gen. nov., sp. nov., isolated from oil-contaminated soil. *Int*. *J*. *Syst*. *Evol*. *Microbiol*. 65(12), 4743-4748. doi: 10.1099/ijsem.0.000643.

Tóth, E.M., Vengring, A., Homonnay, Z.G., Kéki, Z., Spröer, C., Borsodi, A.K., et al. (2014). *Phreatobacter oligotrophus* gen. nov., sp. nov., an alphaproteobacterium isolated from ultrapure water of the water purification system of a power plant. *Int*. *J*. *Syst*. *Evol*. *Microbiol*. 64(Pt 3), 839-845. doi: 10.1099/ijs.0.053843-0.

Tsujisaka, Y., and Mitsuhashi, M. (1993). "Pullulan" in *Industrial Gums (Third Edition)*. Eds. R.L. Whistler and J.N. Bemiller (London: Academic Press), 447-460.

Wang, X.L., and Yao, Y.J. (2011). Host insect species of *Ophiocordyceps sinensis*: a review. *Zookeys* (127), 43-59. doi: 10.3897/zookeys.127.802.

Xia, S., Shi, Y., Fu, Y., and Ma, X. (2005). DGGE analysis of 16S rDNA of ammonia-oxidizing bacteria in chemical-biological flocculation and chemical coagulation systems. *Appl*. *Microbiol*. *Biotechnol*. 69(1), 99-105. doi: 10.1007/s00253-005-0035-5.

Xie, C.-H., and Yokota, A. (2005). *Dyella japonica* gen. nov., sp. nov., a γ-proteobacterium isolated from soil. *Int*. *J*. *Syst*. *Evol*. *Microbiol*. 55(2), 753-756. doi: 10.1099/ijs.0.63377-0.

Xie, J., Strobel, G.A., Feng, T., Ren, H.S., Mends, M.T., Zhou, Z.Y., et al. (2015). An endophytic *Coniochaeta velutina* producing broad spectrum antimycotics. *J*. *Microbiol*. 53(6), 390-397. doi: 10.1007/s12275-015-5105-5.

Xie, L., Chen, Y.L., Long, Y.Y., Zhang, Y., Liao, S.T., Liu, B., et al. (2019). Three new species of *Conlarium* from sugarcane rhizosphere in southern China. *Mycokeys* (56), 1-11. doi: 10.3897/mycokeys.56.35857.

Xu, L.J., Niu, X.Y., Li, X., Zheng, Y.Y., Feng, H.L., Fu, Q., et al. (2022). Effects of nitrogen addition and root fungal inoculation on the seedling growth and rhizosphere soil microbial community of *Pinus tabulaeformis*. *Front*. *Microbiol*. 13. doi: 10.3389/fmicb.2022.1013023.

Yamada, K., Fukuda, W., Kondo, Y., Miyoshi, Y., Atomi, H., and Imanaka, T. (2011). *Constrictibacter antarcticus* gen. nov., sp. nov., a cryptoendolithic micro-organism from Antarctic white rock. *Int*. *J*. *Syst*. *Evol*. *Microbiol*. 61(8), 1973-1980. doi: 10.1099/ijs.0.026625-0.

Yang, J., Liu, J.K., Hyde, K.D., Jones, E.B.G., and Liu, Z.Y. (2018). New species in *Dictyosporium*, new combinations in Dictyocheirospora and an updated backbone tree for Dictyosporiaceae. *Mycokeys* (36), 83-105. doi: 10.3897/ mycokeys.36.27051.

You, C.J., Liu, X., Li, L.X., Tsui, C.K.M., and Tian, C.M. (2017). *Dothiorella magnoliae*, a new species associated with dieback of *Magnolia grandiflora* from China. *Mycosphere* 8(2), 1031-1041. doi: 10.5943/mycosphere/8/2/6.

Zhang, H., Sekiguchi, Y., Hanada, S., Hugenholtz, P., Kim, H., Kamagata, Y., et al. (2003). *Gemmatimonas aurantiaca* gen. nov., sp. nov., a gram-negative, aerobic, polyphosphate-accumulating micro-organism, the first cultured representative of the new bacterial phylum *Gemmatimonadetes* phyl. nov. *Int*. *J*. *Syst*. *Evol*. *Microbiol*. 53(Pt 4), 1155-1163. doi: 10.1099/ijs.0.02520-0.

Zhou, Y., Gong, G.S., Zhang, S.R., Liu, N., Wang, J.J., Li, P.L., et al. (2014). A new species of the genus *Trematosphaeria* from China. *Mycol*. *Prog*. 13(1), 33-43. doi: 10.1007/s11557-013-0889-3.
